# A systematic review on the direct approach to elicit the demand-side cost-effectiveness threshold: Implications for low- and middle-income countries

**DOI:** 10.1371/journal.pone.0297450

**Published:** 2024-02-08

**Authors:** Anh Nu Vu, Minh Van Hoang, Lars Lindholm, Klas Göran Sahlen, Cuc Thi Thu Nguyen, Sun Sun

**Affiliations:** 1 Department of Epidemiology and Global Health, Umeå University, Umeå, Sweden; 2 Department of Health Economics, Hanoi University of Public Health, Hanoi City, Vietnam; 3 Department of Pharmaceutical Management and Economics, Faculty of Pharmaceutical Management and Economics, Hanoi University of Pharmacy, Hanoi City, Vietnam; 4 Department of Learning, Informatics, Management and Ethics, Karolinska Institute, Stockholm, Sweden; Al Mansour University College-Baghdad-Iraq, IRAQ

## Abstract

Several literature review studies have been conducted on cost-effectiveness threshold values. However, only a few are systematic literature reviews, and most did not investigate the different methods, especially in-depth reviews of directly eliciting WTP per QALY. Our study aimed to 1) describe the different direct approach methods to elicit WTP/QALY; 2) investigate factors that contribute the most to the level of WTP/QALY value; and 3) investigate the relation between the value of WTP/QALY and GDP per capita and give some recommendations on feasible methods for eliciting WTP/QALY in low- and middle-income countries (LMICs). A systematic review concerning select studies estimating WTP/QALY from a direct approach was carried out in seven databases, with a cut off date of 03/2022. The conversion of monetary values into 2021 international dollars (i$) was performed via CPI and PPP indexes. The influential factors were evaluated with Bayesian model averaging. Criteria for recommendation for feasible methods in LMICs are made based on empirical evidence from the systematic review and given the resource limitation in LMICs. A total of 12,196 records were identified; 64 articles were included for full-text review. The WTP/QALY method and values varied widely across countries with a median WTP/QALY value of i$16,647.6 and WTP/QALY per GDP per capita of 0.53. A total of 11 factors were most influential, in which the discrete-choice experiment method had a posterior probability of 100%. Methods for deriving WTP/QALY vary largely across studies. Eleven influential factors contribute most to the level of values of WTP/QALY, in which the discrete-choice experiment method was the greatest affected. We also found that in most countries, values for WTP/QALY were below 1 x GDP per capita. Some important principles are addressed related to what LMICs may be concerned with when conducting studies to estimate WTP/QALY.

## Introduction

Due to increasing health expenditures and scarcity in resources, policymakers for health care are facing the challenges of how to allocate health care resources efficiently. Cost-utility analyses have gained popularity in health technology assessments, as they apply quality-adjusted life years (QALYs) as health outcomes, which enables comparisons across different disease and treatment programs [1]. A relevant question would then be how to assign the relevant monetary value to each QALY [2], i.e., how much money are governments willing to spend on additional QALYs? Following this line of thought, it means that based on results from a cost-utility analysis, health technology below a certain national threshold value (cost per QALY) will be considered cost-effective and thus reimbursed [3, 4]. Such information is helpful for better consistency and transparency in reimbursement decisions in health care. As low- and middle-income countries (LMICs) are facing even higher resource scarcities, it becomes even more important for LMICs to have an appropriate threshold value for reimbursement decisions within health care [5].

Threshold values have been established in Europe [3, 6–26], the US [27–36], and a few Asian countries, such as Iran [37–41], Thailand [42–44], Japan [45–47], China [48, 49], and Malaysia [50], but only two studies were conducted in LMICs, including Thailand in 2008 [42] and Vietnam in 2018 [51]. Although World Health Organization (WHO) had no longer recommended a threshold value between 1–3 times the gross domestic product (GDP) per capita per DALY averted [52–54], in countries that lack their own threshold values, this value has still often applied, especially in LMICs [55]. Furthermore, both DALYs and QALYs translate the impact of non-fatal health effects into a life year measure, so that the years of life lived in different health states or lost to premature fatality can be combined into a single indicator [53]. Therefore, in practice, most countries use this value for QALY as well. However, it is quite often argued that the WHO recommendation might lack empirical evidence, and it might lead to inappropriate decisions regarding treatment adoption and resource allocation in health care services [53, 54, 56], as seldom the WTP/QALY exceeds 1 x GDP per capita, if one applies the 2–3 x GDP per QALY, might exhaust the national health budget.

The threshold value varies largely across countries, as health systems and affordability differ [54, 57], and methods for eliciting threshold values also vary considerably [42, 44, 58]; however, thus far, there has been no agreement on which method can be considered the standard method [59]. There are two well-known conceptual perspectives used to derive such threshold values: the supply-side opportunity cost perspective and the demand-side willingness to pay (WTP) perspective [54, 56]. The former perspective focuses on identifying the opportunity cost resulting from the disinvestment required to adopt a new technology [2, 54], while the latter refers to the willingness to pay for a small health gain and then aggregating the WTP needed for a QALY [2, 54]. The supply-side perspective also requires comprehensive and comparable information on the cost per QALY of all interventions and thus is less used in practice relative to the demand-side WTP [41].

For the demand-side WTP, two general approaches are used: 1) directly eliciting individuals’ WTP by using surveys and 2) indirectly inferring a value of health gain by estimating WTP for reductions in mortality or willingness to accept a risk, which is also known as the value of statistical life (VSL) method [2, 59, 60]. To date, most studies have applied the first approach [2, 59].

**Fig 1:**
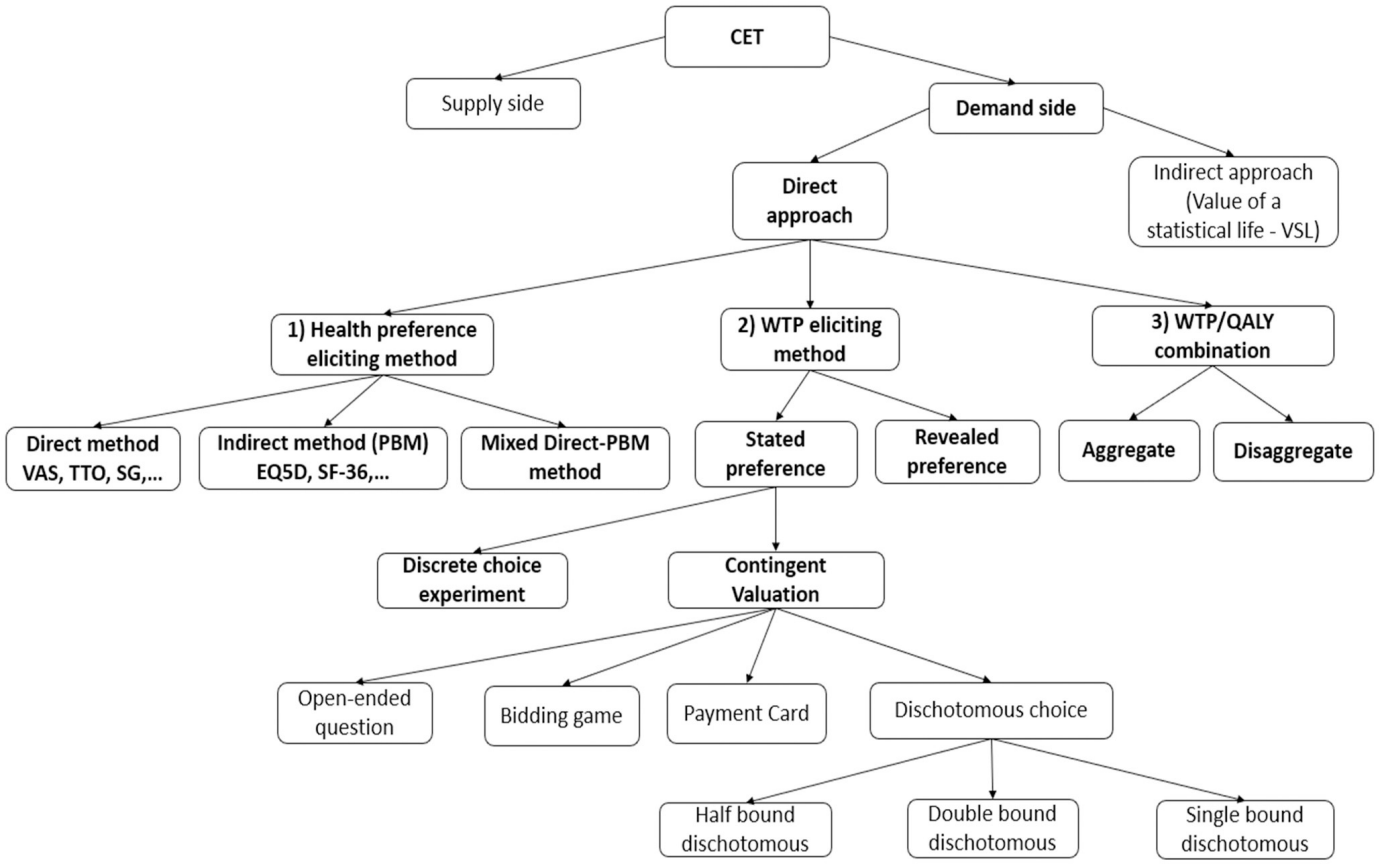
Flow chart for estimating the cost-effectiveness threshold value using a direct approach from the demand-side perspective.

The process of directly eliciting WTP per QALY generally involves three steps (Fig 1): 1) estimating health gain in terms of health preference, 2) eliciting the WTP for that health gain, and 3) combining the estimates from steps 1 and 2 to estimate WTP for a QALY (2). In terms of estimating health gain in step 1, one can elicit health preference by either using a health preference measure (direct method) or via multi-attribute utility measures (indirect method) [61, 62]. The detailed interpretation of Fig 1 is presented in S1 Text.

Several literature review studies have been conducted to evaluate the implementation of different methods [1, 2, 49, 55, 57, 59, 63–66]. However, only a few are systematic literature reviews [49, 57, 59, 64], where the rest are overviews or narrative reviews [1, 2, 55, 60, 63, 65]. Most of these reviews did not investigate the different methods in eliciting threshold values, especially in-depth reviews of the directly eliciting WTP per QALY, which are lacking [1, 55, 56, 63, 64, 66]. Two systematic reviews explored how different methods might impact the threshold value [1, 63, 65]; however, no study applied a regression technique to incorporate all the relevant methodological characteristics simultaneously, and little is known regarding which methodological characteristics are most influential.

This aim of the study is to 1) describe the different methods that have been used for eliciting WTP/QALY with the direct approach; 2) investigate which factors contribute most to the level of values of WTP/QALY; and 3) investigate the relation between the value of WTP/QALY and GDP per capita and give some recommendations regarding which methods might be more feasible for eliciting WTP/QALY in the LMICs.

## Material and methods

### Study design

This systematic review was carried out following PRISMA guidelines [67] to document the knowledge gap regarding how WTP per QALY was elicited, identifying all influential factors.

### Data sources and search strategy

A systematic search with a publication restriction from January 2000 to March 2022 was conducted in seven databases, including PubMed, Embase, Psycinfo, Centre for Reviews and Dissemination (CRD), Cumulative Index to Nursing and Allied Health Literature (CINAHL), EconLit, and International HTA.

Search terms were constructed based on PICOS domains (Population, Intervention, Comparison, Outcomes, and Study design) [68] with O for WTP in combination with QALY. The detailed search strategies are shown in S2 Text. In addition, we also reviewed all references of the included studies in case some eligible studies had not been identified through the search.

### Inclusion and exclusion criteria

Original studies conducted in any country were included if they elicited WTP per QALY in health-related issues by a direct approach. Studies were excluded if they were (i) not available as a full-text paper (available only as an abstract or poster); (ii) not written in English; (iii) just a literature review; or (iv) applying an indirect approach that used VSL.

#### Critical appraisal of studies: Quality assurance process

Two investigators independently performed abstract screening, full-text reviews, information extraction and quality assessment. Disagreements were resolved by consensus in discussion with the rest of research team.

Quality assurance was implemented in four steps: (i) All records identified through database searching were imported into the reference library software Zotero 5.0.92; and, duplicates of these records were excluded by either a merging tool or Zotero. (ii) After removing duplicates, the titles and abstracts of these articles were screened. (iii) The full-text articles were assessed for eligibility to fulfill the selection criteria. (iv) The quality of articles was appraised by using the Appraisal tool for Cross-Sectional Studies (AXIS tool) with 20 components developed by Downes et al. [69] in 2016 in S1 Table. Each question in the AXIS tool was answered as “yes”, “no”, “unclear”, or “not applicable.”

### Information extraction and data preparation

Information on the full text was extracted using a standard extraction form approved by the research group. The details of the extracted information are presented in S3 Text. Moreover, data for gross domestic product (GPD) per capita for each study were also retrieved from the World Bank [70] based on the reporting year (or year of publication if the reporting year was unavailable) and country of study.

### Data analysis

Descriptive analyses were used to describe the extracted data. Continuous variables are expressed as the mean (standard deviation (SD)) and median (interquartile range (IQR)), and for categorical variables, counted frequency and percentage were applied. To compare the threshold value across different countries and time periods, the ratios of WTP per QALY divided by GDP per capita were extracted or estimated if lacking this value. The different currencies were firstly converted to US dollars using the exchange rate in the reporting year, and then converted to international dollars (i$) values in 2021 by using the country’s consumer price index (CPI) [71] and purchasing power parity (PPP) [71, 72]. The Kruskal‒Wallis analysis was applied to test the WTP per QALY differences between category groups.

To evaluate which factors could influence WTP per QALY, the Bayesian Model Averaging (BMA) method was applied to select candidate covariates. The BMA approach could address the uncertainty in the variable selection process by selecting a number of all possible models and performing all inferences and predictions via the posterior probabilities of these models [65, 73]. The model with the lowest Bayesian information criterion (BIC) and the highest posterior probability was the best selected model [74]. The factors were assessed, including year of publication, reporting year, continent, number of scenarios, options of scenarios, subjects, mode of administration, number of WEM, number of UEM, kind of WEM and kind of UEM.

All statistical analyses were performed in R version 4.0.0, and a p value < 0.05 was considered statistically significant.

### Criteria for recommendation for feasible methods in LMICs

The recommendations are made based on 1) empirical evidence from the systematic review, which method might be most scientifically approved and applied; and 2) given the resource limitation in LMICs, which methods are most feasible in terms of data availability within the budget constraints.

## Results

### Study selection

**Fig 2:**
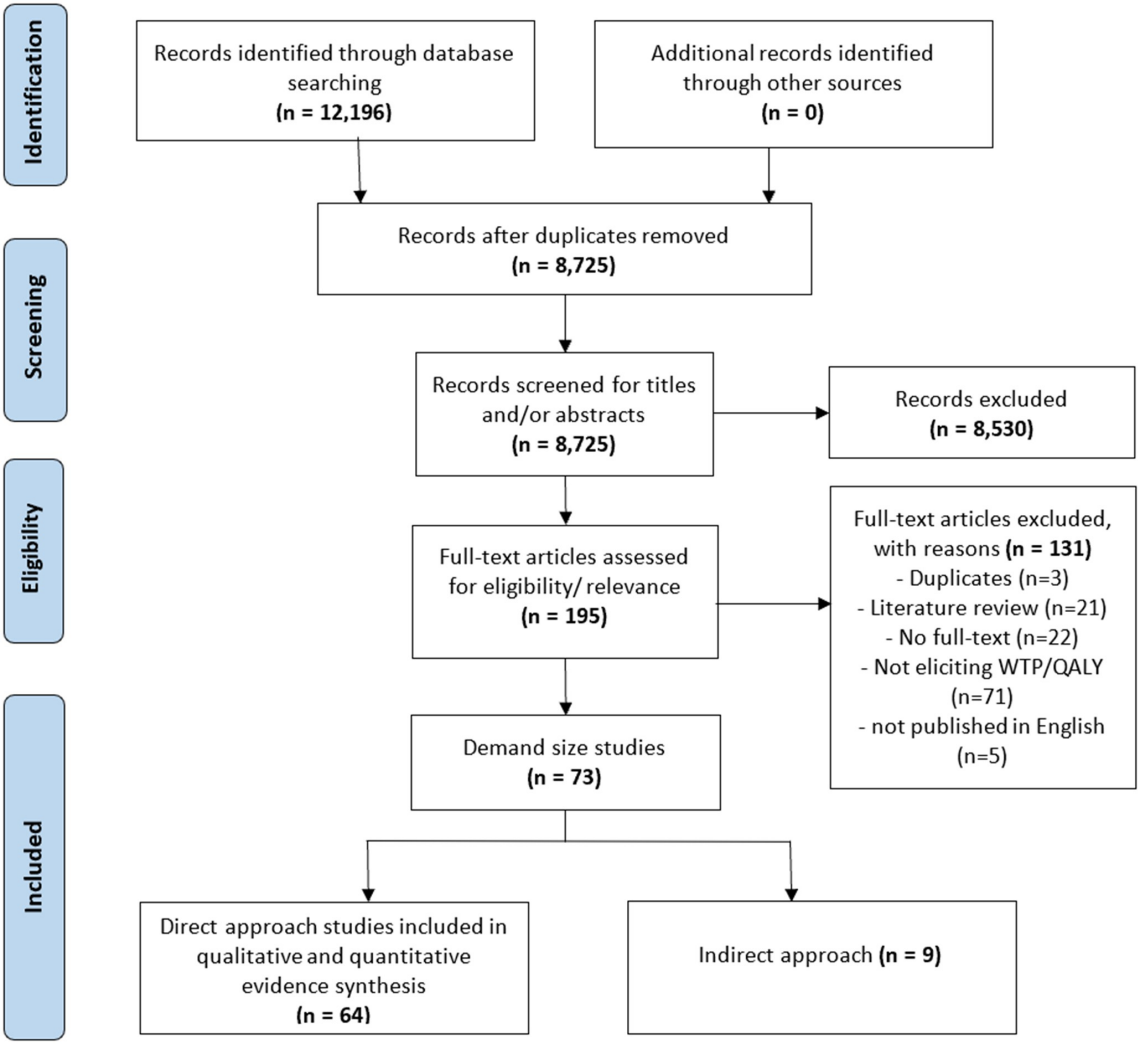
PRISMA flow diagram of article identification and selection procedure.

The study selection process is presented as a PRISMA flow diagram in Fig 2. The search terms in the seven databases yielded a total of 12,196 records, and 3,471 records were removed due to duplication, leaving 8,725 records for title and abstract screening. Based on the inclusion/exclusion criteria, 8,530 records were excluded. In total, 195 articles were reviewed as full-text, among which 131 were excluded for the following reasons: duplicated (n = 3), not eliciting WTP/QALY value (n = 71), literature reviews (n = 21), not available in full text (n = 22), not in English (n = 5), and indirect approach (n = 9). Overall, 64 articles were used for data extraction.

### Study characteristics

#### General characteristics of the studies

The study characteristics are reported in Table 1. The results from the review suggested that most articles (82.8%) were published after 2010; the number of publications conducted in the five years from 2015 to 2020 was equal to the total number of those published before 2015. Studies were mostly from Europe (48.4%) and Asia (35.9%). More than 70% of the studies were from high-income countries, nearly 30% were from middle-income countries (upper middle-income countries-27%, lower middle-income countries-3%), and no study was found in low-income countries. The majority restricted the scope to within a country (95.3%), and only three studies (4.7%) were conducted in multiple countries. Most studies had a first author affiliation from universities (71.9%), funding sources (70.3%), and no conflict of interest (67.2%).

**Table 1:** Overview of study characteristics.

Study Characteristics	Count	%	Median (IQR)	Mean (SD)	p—value
Total number or articles reviewed	**64**				
Publication year					
Before 2000	1	1.6%	18,460.7 (0.0)	18,460.7 (NA)	<0.001
During 2000–2005	4	6.3%	14,462.6 (26,013.2)	19,652.7 (15,535.8)	
During 2006–2010	6	9.4%	57,264.2 (41,462.5)	66,173.5 (40,778.1)	
During 2011–2015	17	26.6%	23,658.9 (50,892.0)	56,211.4 (77,915.5)	
During 2016–2020	29	45.3%	7,092.4 (22,029.6)	29,599.1 (81,170.9)	
2021—now	7	10.9%	8,273.8 (20,820.1)	20,268.5 (27,606.4)	
Reporting year					
Before 2000	1	1.6%	18,460.7 (0.0)	18,460.7 (NA)	0.285
During 2000–2005	4	6.3%	20,032.7 (33,315.2)	24,723.8 (18,749.5)	
During 2006–2010	16	25.0%	23,167.0 (40,140.1)	34,468.0 (35,368.1)	
During 2011–2015	14	21.9%	10,753.4 (37,496.6)	33,810.4 (59,303.1)	
During 2016–2020	16	25.0%	9,791.9 (33,539.7)	36,987.8 (79,413.0)	
2021—now	0	0.0%			
*not reported*	13	20.3%	23,095.8 (45,597.9)	63,384.4 (115,310.9)	
Region					
Europe	31	48.4%	22,750.9 (33,316.4)	54,841.9 (99,413.6)	<0.001
The US	9	14.1%	7,649.4 (30,669.2)	20,018.6 (26,759.4)	
Asia	23	35.9%	8,881.6 (42,905.4)	27,929.8 (35,861.8)	
Australia	1	1.6%	65,444.8 (22,600.8)	61,093.0 (22,662.6)	
Number of countries per study					
1	61	95.3%	12,998.8 (31,399.0)	36,781.4 (73,188.0)	<0.001
>1	3	4.7%	51,600.7 (48,612.9)	57,232.6 (30,357.4)	
*not reported*	0	0%			
Type of country income					
High-income	46	71.9%	24,246.7 (44,547.0)	48,086.7 (79,955.8)	<0.001
Middle-income	18	28.1%	6,306.6 (6,690.8)	15,310.1 (28,201.9)	
Upper middle-income	16	25.0%	5,936.4 (7,233.6)	16,306.6 (30,052.3)	
Lower middle-income	2	3.1%	7,422.3 (3,879.8)	8,641.0 (5,808.6)	
Low-income	0	0%			
First author affiliation					
Academic/university	55	85.9%	10,824.0 (29,837.1)	32,220.8 (62,446.2)	<0.001
Research agency/group	3	4.7%	53,074.8 (119,509.3)	128,074.1 187,989.6)	
Government institution	22	3.1%	58,547.6 (32,163.0)	72,920.3 (72,508.6)	
*not reported*	4	6.3%	34,223.5 (50,936.6)	58,251.1 (59,184.6)	
Funding source reported					
Declared funding source	45	70.3%	19,929.2 (39,975.6)	42,383.3 (75,783.6)	<0.001
Declared no funding source	3	4.7%	2,643.6 (5,875.4)	10,457.3 (17,371.2)	
Did not report funding source	11	17.2%	3,306.0 (9,210.7)	12,333.3 (29,853.3)	
*not reported*	5	7.8%	34,223.5 (50,936.6)	58,251.1 (59,184.6)	
Conflict of interest reported					
Reported conflict of interest	2	3.1%	34,864.2 (19,154.7)	34,859.8 (32,665.0)	<0.001
Reported no conflict of interest	43	67.2%	9,905.2 (27,120.7)	36,601.3 (80,453.8)	
Did not report conflict of interest	14	21.9%	23,463.6 (59,292.5)	40,745.6 (46,173.5)	
*not reported*	5	7.8%	34,223.5 (50,936.6)	58,251.1 (59,184.6)	

#### Characteristics of the research design

The characteristics of the method related to eliciting WTP per QALY are reported in Table 2. Individual perspectives were mostly used (78.1%), followed by societal perspectives with exclusive or inclusive individual ones (9.4%). Most studies used collected data from the general population (70.3%), face-to-face interviews (40.6%) or web-based surveys (39.1%). The sample size was mostly over 1000 participants, followed by 100–500 people.

**Table 2:** Overview of the methods for eliciting willingness to pay per quality-adjusted life year.

Research Methodology	Count	%	Median (IQR)	Mean (SD)	p—value
*Total number or articles reviewed*	**64**				
Perspectives					
Individual	50	78.1%	12,132.2 (27,122.8)	30,418.8 (67,419.4)	<0.001
Societal	6	9.4%	4,550.1 (42,765.8)	66,363.3 (113,964.6)	
Healthcare provider	2	3.2%	139,160.0 (49,701.7)	132,013.3 (43,922.8)	
Family member of patient	2	3.2%	36,292.1 (36,395.4)	40,221.2 (31,721.5)	
Individual and societal	3	4.8%	65,763.1 (38,396.3)	69,932.2 (29,138.8)	
Individual and healthcare provider	1	1.6%	63,433.7 (108,232.3)	78,664.9 (62,780.3)	
Study sample					
General population	45	70.3%	16,232.4 (37,897.0)	39,262.0 (75,160.9)	<0.001
Patients	8	12.5%	11,376.3 (27,177.0)	18,053.4 (16,050.9)	
Clinicians	2	3.1%	139,160.0 (49,701.7)	132,013.3 (43,922.8)	
General population and patients	7	10.9%	9,819.7 (12,484.2)	16,319.1 (17,894.8)	
Both clinicians and politicians	1	1.6%	63,433.7 (108,232.3)	78,664.9 (62,780.3)	
Family member of patients	1	1.6%	48,389.5 (28,227.2)	51,077.8 (28,323.0)	
Sample size					
<100	1	1.6%	145,833.8 (2,065.0)	145,833.8 (2,920.4)	<0.001
100–500	22	34.4%	7,649.4 (21,692.7)	21,088.5 (32,703.2)	
501–1000	15	23.4%	9,839.8 (26,595.4)	41,359.8 (94,688.4)	
>1000	24	37.5%	23,770.9 (47,533.1)	46,079.1 (74,052.2)	
*not reported*	2	3.1%	24,388.4 (50,763.0)	52,056.3 (54,266.3)	
Mode of administration					
Face-to-face interview	26	40.6%	7,534.7 (19,202.2)	21,670.0 (48,232.8)	<0.001
Telephone	4	6.3%	16,647.6 (45,167.9)	33,309.7 (35,697.1)	
Web-based survey	25	39.1%	30,527.6 (58,389.4)	56,620.9 (93,486.9)	
Self-administered questionnaire	5	7.8%	4,945.7 (16,794.4)	16,169.0 (19,975.3)	
Secondary data analysis	2	3.1%	21,025.1 (54,414.6)	47,171.2 (57,429.4)	
Other combination	2	3.1%	47,700.0 (52,220.2)	65,367.8 (61,362.9)	
Number of hypothetical scenarios					
1	12	18.8%	12,438.9 (24,859.2)	19,399.5 (15,818.1)	0.186
2–5	20	31.3%	13,135.9 (55,498.3)	41,359.5 (79,131.7)	
6–10	14	21.9%	12,001.7 (29,925.4)	32,719.6 (56,574.4)	
>10	14	21.9%	46,182.5 (85,315.2)	19,733.9 (44,865.7)	
*not reported*	4	6.3%	27,409.4 (20,276.1)	41,213.0 (41,445.7)	
Context of hypothetical scenario					
Ex post	24	37.5%	18,608.7 (37,280.6)	32,904.8 (51,311.5)	<0.001
Ex ante	27	42.2%	23,597.3 (49,298.9)	52,483.6 (89,508.8)	
Both ex post and ex ante	9	14.1%	3,729.6 (4,228.2)	7,645.8 (12,477.2)	
Not applicable/not reported	4	6.3%	14,912.0 (23,775.5)	36,578.6 (52,165.9)	
Type of hypothetical scenario					
Specific	20	31.3%	5,902.5 (23,280.9)	33,211.5 (76,081.7)	<0.001
Not specific to any diseases/illness	40	62.5%	21,727.6 (44,543.2)	43,517.5 (70,455.1)	
Both specific and current health state	2	3.1%	6,432.7 (4,745.7)	6,416.2 (3,041.8)	
Not applicable/not reported	2	3.1%	9,019.1 (12,517.4)	12,885.3 (10,496.3)	
Type of QALY gain					
Improving quality of life	39	60.9%	12,981.5 (27,835.8)	32,685.7 (67,062.7)	<0.001
Extending life	2	3.1%	13,675.0 (10,061.5)	129,272.8 (267,984.0)	
Life saving	3	4.7%	73,625.4 (36,183.4)	65,092.1 (34,315.8)	
Improving quality of life and extending life	8	12.5%	26,233.2 (78,681.6)	58,990.7 (79,857.7)	
Improving quality of life, extending life and saving life	8	12.5%	23,659.0 (38,710.4)	31,343.3 (27,026.1)	
Others	2	3.1%	4,550.1 (4,489.9)	6,025.6 (4,900.8)	
Not applicable	2	3.1%	9,019.1 (12,517.4)	12,885.3 (10,496.3)	
Informed QALY gain					
Informed QALY gain	20	31.3%	32,490.1 (61,889.7)	56,353.9 (77,899.7)	<0.001
Uninformed QALY gain	41	64.1%	9,560.7 (25,882.4)	27,823.4 (64,409.0)	
Not applicable	3	4.7%	9,019.1 (10,689.6)	10,300.4 (6,522.4)	
Duration of hypothetical scenario					
< 1 month	1	1.6%	23,385.4 (74,462.6)	80,330.0 (125,829.0)	<0.001
1 month– 1 year	25	39.1%	6,663.2 (15,037.0)	12,984.7 (13,664.3)	
> 1 year	19	29.7%	26,400.5 (50,101.4)	39,820.4 (39,931.8)	
Both duration	15	23.4%	9,019.1 (12,517.4)	12,885.3 (10,496.3)	
Not applicable/not reported	4	6.3%	588.2 (2,418.6)	8,557.1 (20,555.1)	
Payment vehicle					
Pay lump sum	22	34.4%	19,071.4 (40,708.7)	32,349.4 (36,448.0)	<0.001
Pay in installments	19	29.7%	24,770.8 (35,258.3)	81,766.3 (135,754.8)	
Both pay lump sum and pay in installments	1	1.6%	20,133.2 (8,882.3)	20,435.0 (6,776.1)	
Pay through taxes and in installments	2	3.1%	2,776.1 (12,602.4)	13,639.5 (20,435.1)	
Not clearly stated	14	21.9%	4,406.3 (22,062.1)	19,163.5 (31,272.7)	
None	6	9.4%	22,665.0 (77,924.9)	52,063.8 (60,732.9)	
Regression analysis					
Yes	51	79.7%	13,307.7 (38,086.0)	38,198.7 (74,627.0)	0.059
No	9	14.1%	28,748.7 (36,586.6)	40,987.1 (50,208.0)	
*not reported*	4	6.3%	29,261.1 (20,627.2)	36,567.8 (25,409.4)	

Regarding scenarios, most studies selected 2 to 5 scenarios (31.3%). The ex-ante context of the hypothetical scenario, which asked how much participants not yet suffering from an illness would pay to lower their risks, is more likely to be used than the ex-post context, which asked respondents already suffering from an illness to pay for specific treatment (42.2% versus 37.5%). The type of hypothetical scenario labelled as unspecified disease/illness was the most used (62.5%). The common type of QALY gain was improving quality of life (60.9%) with unfixed/closed value gain in which respondents did not know the size of the gain (66.1%). The most popular duration of the hypothetical scenario is the period from 1 month to 1 year (39.1%). In addition, 34.4% of the studies used lump-sum payments. Nearly 80% of the studies used regression analysis to analyze the influencers on WTP per QALY value.

#### Characteristics of methods to elicit health preference

Details regarding the methods used for eliciting health preferences are reported in Table 3. Methods for eliciting preference vary largely across studies, among which the directly elicited health preference methods were mostly applied (43.8%), followed by the indirectly elicited health preference methods which are known as the preference-based quality of life measures (PBM) (34.4%). Among the direct methods (standard gamble (SG), time trade-off (TTO), and the visual analog scale (VAS)), the majority applied mixed methods (10 out of 27, 37.0%) and VAS (9 out of 27, 33.3%); and among the PBM, the majority applied the EQ-5D instrument (19 out 22 studies, 86.4%). It is difficult to tell whether 3L or 5L was more popular, as 7 studies did not report which EQ-5D version was applied. Among those that applied the EQ-5D instrument, most studies (13 out of 19) applied both the EQ-5D index and EQ VAS; however, a few studies (7 out of 19) presented both values. Among the 6 studies that also used mixed methods, the mix types varied and were heterogeneous because no studies used the same mix method.

**Table 3:** Reporting methods for eliciting health preference in relation to estimating willingness to pay per quality-adjusted life year.

Health preference eliciting methodology	n = 64	%	Median (IQR)	Mean (SD)
**Methods for directly eliciting health preference**	28	43.8%	7,705.6 (24,170.3)	33,041.6 (77,241.8)
SG	2	3.1%	38,572.4 (27,204.4)	61,130.7 (64,302.3)
TTO	6	9.4%	19,094.9 (35,477.3)	24,565.5 (23,494.1)
VAS	9	14.1%	14,197.4 (31,669.4)	89,716.5 (162,886.5)
Person Trade-Off (PTO)	1	1.6%	2,045.0 (1,263.1)	2,005.7 (984.9)
**Mixed methods**	10	15.6%	4,532.3 (13,874.4)	16,512.8 (34,445.2)
SG and TTO	1	1.6%	34,864.2 (12,326.9)	33,157.2 (7,747.4)
SG or TTO and VAS	2	3.1%	23,328.6 (53,207.1)	57,677.3 (65,117.9)
TTO and VAS	2	3.1%	3,585.7 (3,149.2)	4,558.6 (4,483.1)
VAS and SG	2	3.1%	437.5 (1,409.8)	1,468.3 (3,027.6)
VAS and SG and TTO	1	1.6%	6,432.7 (2,251.4)	6,403.3 (2,534.5)
TTO and rating scales	2	3.1%	16,686.4 (1,774.3)	16,686.4 (2,509.3)
**Methods for indirectly eliciting health preference (PBM)**	23	35.9%	19,375.3 (45,989.2)	41,701.2 (79,251.6)
**EQ-5D instrument**	19	29.7%	19,644.9 (50,078.4)	48,857.4 (87,011.5)
*EQ-5D-3 L*	9	14.1%	8,177 (16,371.8)	17,441.4 (19,955.8)
Either EQ-5D index value or EQ VAS scores was used, but not specified in the study	2	3.1%	8,177.2 (4,591.8)	8,044.6 (3,763.9)
EQ-5D index	3	4.7%	52,057.0 (26,026.4)	40,224.7 (22,891.0)
Both EQ-5D index value and EQ VAS scores were used and reported	4	6.3%	2,643.6 (17,916.5)	11,485.5 (16,044.1)
*EQ-5D-5 L*	3	4.7%	38,050.5 (48,392.6)	73,505.6 (68,829.6)
Either EQ-5D index value or EQ VAS scores was used, but not specified in the study	2	3.1%	11,879.0 (45,684.1)	30,235.8 (37,009.7)
EQ-5D index	1	1.6%	228,214.8 (51,265.1)	228,214.8 (72,499.8)
*EQ-5D (not specified 3 L or 5 L)*	7	10.9%	38,050.5 (48,392.6)	73,505.6 (117,087.4)
Either EQ-5D index value or EQ VAS scores was used, but not specified in the study	2	3.1%	14,667.0 (9,126.1)	15,879.6 (7,212.8)
EQ-5D index	2	3.1%	57,130.3 (34,561.6)	51,684.1 (23,504.9)
Both EQ-5D index value and EQ VAS scores were used and reported	3	4.7%	80,931.2 (296,218.5)	185,155.7 (202,288.5)
**SF-6D**	2	3.1%	26,725.0 (6,298.1)	26,361.5 (4,113.7)
**Mix of PBM**	2	3.1%	8,220.7 (4,335.4)	7,299.7 (3,309.3)
Quality of Well-being Scale-self-administered version (QWB-SA) and EQ-5D	1	1.6%	6,594.2 (7,135.1)	6,445.2 (4,458.5)
EQ-5D and SF-6D	1	1.6%	8,220.7 (3,196.4)	8,154.3 (1,930.8)
**Mix of the direct elicited health preference method and PBM**	6	9.4%	20,813.6 (24,328.3)	21,897.1 (3,309.3)
EQ-5D and TTO	3	4.8%	19,012.9 (22,952.5)	19,765.7 (13,072.9)
EQ-5D-3L and TTO and SG	1	1.6%	6,186.4 (0.0)	6,186.4 (NA)
SF36 and SF12 and SG and TTO and VAS	1	1.6%	33,437.0 (12,520.5)	34,386.3 (10,338.1)
Other	1	1.6%	8,246.3 (5,387.0)	9,378.8 (6,745.1)
**Not reported**	7	10.9%	52,202.2 (71,980.5)	61,849.2 (49,376.0)

### Characteristics of the willingness to pay-eliciting method

Details regarding how the WTP questions are addressed are reported in Table 4. The majority of studies applied the contingent valuation method (89.1%), among which most mixed more than two approaches (28 out of 57 studies), usually either a bidding game or an open-ended question with other approaches. For studies that applied only one approach, the bidding game (n = 7) and double-bound dichotomous choice question (n = 7) were mostly used.

**Table 4:** Willingness to pay eliciting methods used by the studies.

Willingness to pay eliciting methodology	Count (n = 64)	%	Median (IQR)	Mean (SD)	*p value*
Revealed preference	2	3.1%	9,019.1 (12,517.4)	12,885.2 (10,496.2)	0.026
Stated preference	62	96.9%	16,689.2 (39,340)	39,067(71,163.8)	
Discrete-choice experiment	5	7.8%	31,127.2 (129,125.0)	92,844.7 (109,421.2)	0.009
Contingent valuation	57	89.1%	15,497.9 (38,272.6)	36,412.0 (67,888.1)	
Open-ended (OE)	6	9.4%	8,023.8 (13,691.6)	33,503.5 (54,632.0)	<0.001
Close-ended	1	1.6%	36,053.1 (40,897.3)	65,223.6 (70,264.8)	
Bidding game (BG)	7	10.7%	2,908.7 (19,765.1)	13,331.0 (17,023.1)	
Card sorting (CS)	2	3.1%	6,694.9 (550.4)	6,722.8 (550.9)	
Payment card	4	6.3%	16,730.8 (24,392.2)	47,165.4 (126,939.4)	
Single-bound dichotomous choice (SBDC)	2	3.1%	74,439.9 (101,763.9)	114,908.8 (129,626.4)	
Double-bound dichotomous choice (DBDC)	7	10.9%	55,376.4 (45,042.1)	55,565.4 (33,061.1)	
Mixed method	28	43.8%	11,081.7 (27,901.9)	30,420.4 (66,348.2)	
BG and DBDC	1	1.6%	12,668.2 (3,564.2)	12,668.2 (5,040.5)	
BG and Payment cards	1	1.6%	34,018.2 (5,269.5)	34,018.2 (7,452.2)	
BG, followed by OE	5	7.8%	8,814.0 (19,233.1)	19,307.1 (21,504.6)	
CS, followed by OE	2	3.1%	36,480.4 (11,722.1)	34,659.0 (7,842.2)	
Payment scale, followed by OE	9	14.1%	3,804.6 (16,930.7)	36,511.6 (106,748.2)	
DBDC, followed by OE	3	4.7%	52,057.0 (44,960.1)	34,488.1 (25,386.4)	
PC, followed by OE	5	7.8%	17,990.2 (34,396.8)	34,240.2 (45,221.4)	
Others	2	3.1%	4,550.0 (4,489.9)	6,025.5 (4,550.0)	

Abbreviations: BG, Bidding game; CS, card sorting; DBDC, double-bound dichotomous choice; OE, open-ended; PC, payment card; SBDC, single-bound dichotomous choice.

#### Characteristics of the WTP/QALY combination method

Table 5 shows the characteristics of the WTP/QALY combination method. Approximately one-third of the studies used the aggregated method to combine WTP per QALY. Moreover, 28.1% applied the disaggregated method, 7.8% combined both the aggregated and disaggregated methods, and approximately 10.9% applied the regression method. Approximately, 15.6% of the studies did not state which method they applied as a combination method.

**Table 5:** Combination method to estimate willingness to pay per quality-adjusted life year.

WTP/QALY combination method	Count (n = 64)	%	Median (IQR)	Mean (SD)	*p—value*
Aggregated	20	31.3%	18,562.8 (34,337.6)	29,122.0 (47,572.8)	<0.001
Disaggregated	18	28.1%	19,581.3 (44,930.8)	39,375.8 (66,885.8)	
Combined aggregated and disaggregated	5	7.8%	5,036.5 (7,039.3)	9,180.1 (10,028.4)	
Regression	7	10.9%	20,813.6 (42,972.5)	59,108.2 (118,156.9)	
Others	4	6.3%	9,791.9 (146,618.9)	87,648.0 (131,761.8)	
Not clearly stated	10	15.6%	13,675.0 (54,952.9)	45,029.3 (61,074.3)	

### Results of WTP per QALY

The results for WTP per QALY by study, country and year are reported in S2 Table. For an overview and easy comparison, WTP per QALY by country after conversion into international dollars in 2021 (i$) [75] was calculated, presented in S3 Table. In general, the median WTP per QALY of countries varied significantly from i$2,643.6 to i$145,833.8, with the lowest in Greece and the largest value in Bulgaria. The study in Bulgaria interviewed doctors, with metastatic cancer as a hypothetical scenario; hence, this resulted in a high WTP/QALY value and high ratio of WTP per QALY per GDP per capita [6]. However, the median WTP per QALY for all countries was i$16,647.6, while the median WTP per QALY per GDP per capita of all studies was 0.534.

**Fig 3:**
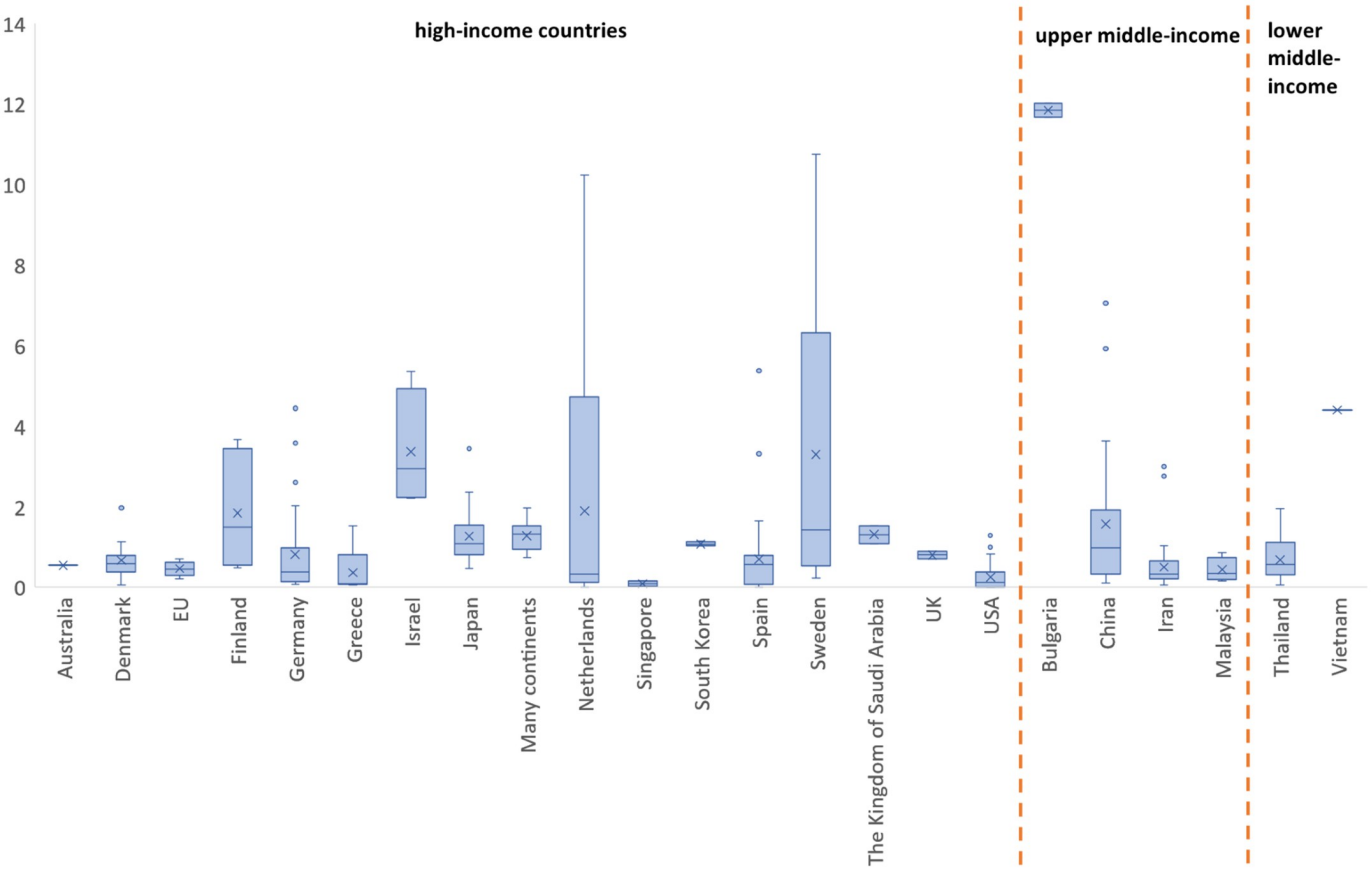
Willingness to pay per quality-adjusted life year per gross domestic product per capita by country.

To reference the WTP per QALY values per GDP per capita of each country, a boxplot chart is shown (Fig 3). Fig 3 demonstrates that most countries had median values under 1 x GDP per capita, with the Bulgaria (11.852), Israel (2.956), Vietnam (4.403) as exceptions. This pattern was observed across high-income (0.557, IQR = 0.935), upper middle-income (0.429, IQR = 0.712) and lower middle-income countries (0.603, IQR = 0.315).

**Fig 4:**
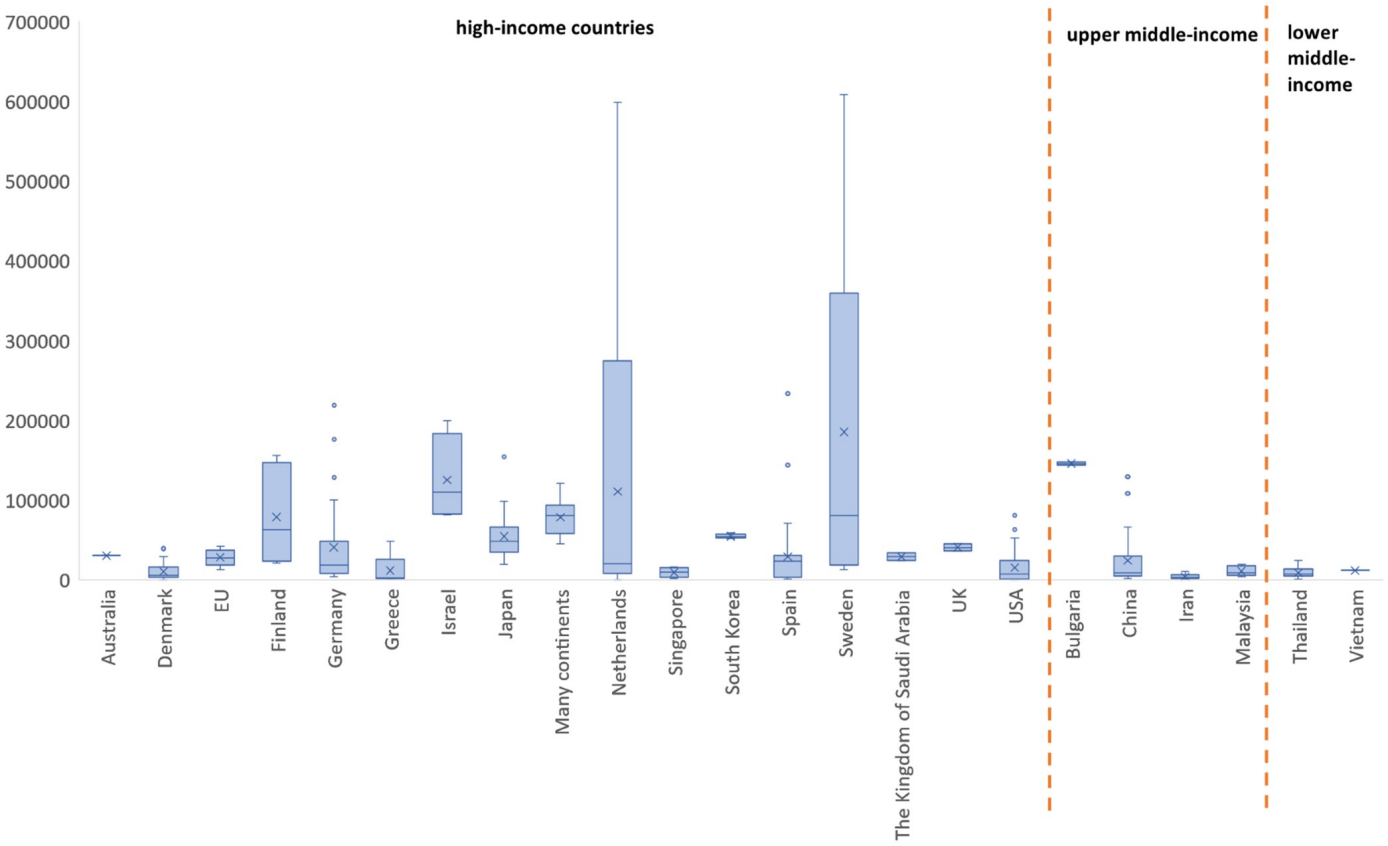
Boxplot of WTP per QALY (i$) by country.

Fig 4 demonstrates the boxplot diagram of willingness to pay per QALY by country converted into the 2021 international dollar. Sweden, the Netherlands, Finland and Israel had a large variability in WTP/QALY results. However, the median WTP/QALY of all countries was generally below 150,000.

### Influential factors to WTP/QALY

#### Subgroup analysis

The detailed results of the subgroup analysis are reported in S4 Text. In general, there were differences in WTP per QALY values between subgroups, and the difference was statistically significant.

#### Multivariate regression analysis

The results from the multivariate analyses are reported in Table 6. Among the 169 evaluated models from BMA, Model 1 is the best model (BIC = -21.43, post probability = 0.046). The influential factors are type of country income (lower middle-income), type of QALY gain (a combination of improving quality of life, extending life, saving life, others or not applicable), context of hypothetical scenario (both ex post and ex ante), duration of hypothetical scenario (>1 year), sample size (501–1000, >1000, not reported), mode of administration (other combination), type of willingness to pay (discrete), specific willingness to pay eliciting methodology (DBDC), payment vehicle (none, not clearly stated) and utility elicitation method (EQ-5D and TTO; EQ-5D-3L index; both the EQ-5D-3L index value and EQ VAS score are used, either the EQ-5D-5L index value or EQ VAS score was used but not specified in the study; SF-6D; combination of VAS/SG/ TTO). The factors that had the posterior probability, or probability that the variables affected the mean willingness to pay per QALY of 100%, are important to the discrete-choice experiment method. The factors had a negative value and thus an opposite direction effect on the WTP/QALY value; conversely, the factors with positive value had the same direction effect. This model explained 34.1% (r^2^ = 0.341) of the difference in the variance of the mean willingness to pay per QALY.

**Table 6:** Results of BMA analysis.

		Posterior probability	Model 1	Model 2	Model 3	Model 4	Model 5
Intercept		100	57,936	48,738	83,748	57,702	48,545
Type of country income	Lower middle-income	96.9	-113,255	-109,097	-138,868	-119,060	-114,952
Type of QALY gain	Saving life	17	.	.	.	-41,450	-41,671
	Improving quality of life and extending life	40.5	-36,106	-34,566	.	-47,511	-46,039
	Improving quality of life, extending life and saving life	66.9	-57,037	-54,914	.	-72,036	-70,002
	Others	68.3	-176,665	-173,591	.	-191,621	-188,640
	Not applicable	88.5	-111,126	-109,197	-115,768	-119,243	-117,366
Context of hypothetical scenario	Both ex post and ex ante	99.8	-67,200	-66,266	-73,425	-72,955	-72,056
Type of hypothetical scenario	Not specific to any diseases/illness	43.1	.	.	(-64,164)	.	.
Duration of hypothetical scenario	>1 year	92.7	-71,067	-69,971	-51,495	-72,207	7–1,123
Sample size	501–1000	88.8	65,081	71,467	64,084	72,816	79,214
	>1000	97.4	61,528	66,683	58,327	67,932	73,098
	Not reported	64.1	83,672	87,803	.	98,093	102,282
Mode of administration	Other combination	46	-54,457	-51,667	.	-59,822	-57,074
Health preference eliciting methodology	Discrete-choice experiment	100	99,913	99,368	103,268	99,961	99,419
WTP eliciting methodology	CS, followed by OE	44.6	.	.	75,749	.	.
	DBDC (double-bound dichotomous choice)	85.4	45,919	48,240	37,602	65,611	68,026
Payment vehicle	None	60.8	105,231	109,439	.	118,099	122,357
	Not clearly stated	98.7	-50,165	-45,648	-69,007	-50,228	-45,733
Utility elicitation method	EQ-5D and TTO	62.4	-68,645	-63,889	.	-77,640	-72,954
	EQ-5D-3L index	93.8	-85,799	-82,399	-59,567	-88,741	-85,373
	Both EQ-5D-3L index value and EQ VAS score are used	43.3	-62,351	-57,288	.	-54,312	-49,228
	Either EQ-5D-5L index value or EQ VAS score was used, but not specified in the study	90.8	93,152	96,807	128,974	94,808	98,456
	SF-6D	88.2	-95,710	-93,389	-109,319	-103,554	-101,285
	VAS and SG and TTO	61	-51,533	.	-77,345	-51,299	.
nVar			21	20	15	22	21
r2			0.341	0.329	0.27	0.35	0.339
BIC			-21.43	-21.34	-20.98	-20.68	-20.56
post prob			0.046	0.044	0.037	0.032	0.03

#### Quality assessment

Results of the quality appraisal of studies using the AXIS tool were presented in S4 Table. All studies defined clearly the objective and target population and had appropriate study designs. Most studies appropriately measure the value of WTP per QALY (96.9%) by using the instruments that had been piloted or published previously (92.2%). Most of them described sufficiently their method (98.4%) and statistical significance (89.1%). However, very few studies adjusted the sample size (9.4%) and non-responders (6.3%). Regarding the results, most studies described adequately the data on WTP and health preference (98.4%), results for analyses (95.3%), and limitations (81.3%). More than half of the studies (65.6%) reported that the study results were not affected by funding sources or conflicts of interest.

## Discussion

We found that the methods for deriving WTP/QALY vary largely across studies, which is consistent with previous findings [59, 64]. The societal perspective, perspective of healthcare provider, type of QALY gain of extending life, and the context of hypothetical scenarios concerning both ex post and ex ante contribute the most to the level of values of WTP/QALY. We also found that in most countries, values for WTP/QALY were below 1 x GDP per capita.

In the following sections, we address some important principles related to what LMICs may be concerned about when conducting studies to estimate WTP/QALY. To begin, relative to the supply-side approach, LMICs may contemplate the adoption of a demand-side direct approach (WTP/QALY), as a means to establish a national threshold value. Several justifications underlie this choice: Firstly, in the past decade, several HICs have focused on the supply-side approach, such as England [63], Spain [76], Sweden [77], The Netherlands [78], Australia [79] which may be more relevant to inform decision making on resource [63, 80]. However, this approach necessitates the availability of substantial and comparable datasets within the health sector, encompassing data for healthcare expenditure and health outcomes, alongside variables to control for healthcare necessity [80, 81]. Regrettably, such comprehensive data is often scarce within LMICs, rendering the demand-side approaches more operationally viable [63]. Secondly, the demand-side approach assumes that the health budget is not finite but fluctuates with response to changing healthcare requirements [54]. This assumption aligns more closely with real-world dynamics, as the health care budget can be compensated by the state budget when it faces deficits.

Among the demand-side WTP methods, the indirect approach using VSL also requires sufficient data on employment and workplace fatalities, which may also not be available in LIMCs. Moreover, the VSL method involves scenarios with a very small reduction in mortality, which can derive higher thresholds relative to the WTP/QALY direct approach [59, 82]. Therefore, it might be more feasible for LMICs to establish the national threshold by using the direct approach for the demand-side method (estimating WTP/QALY). However, it is crucial to carefully consider methodological rigor, generalizability, and ethical implications in order to ensure the validity and applicability of the results. It requires collaborative efforts that involve policymakers, researchers, and stakeholders to establish robust and widely accepted cost-effectiveness thresholds using the direct approach in LMICs.

### Perspective

Most studies (78.1%) applied the individual perspective, where the respondents made the choice that maximizes his/her own benefit along the principles in Welfarism. However, we, like Bobinac et al. (2013), judge the theoretical reasons for a societal perspective more convincing. The social value of a QALY is defined as the amount of consumption that individuals are willing to forego to contribute to a health gain achieved in society. This gain may, or more frequently may not, accrue to the payer. We thus think that social value is the most reasonable construct in a society with collectively funded health care. Citizens pay regularly regardless of whether they at any particular point in time need health care, and the incremental cost at the time point of consumption of health care is relatively small.

### Population

Most studies (70.3%) used the general population as the study sample, as it contains a heterogeneous population, and the results can, based on this, be generalized [83]. A smaller number of studies used patients, clinicians or politicians as respondents, which limits the findings to certain health conditions [42], hence the results might not be generalizable to other population groups. Therefore, the recommendation of using the general population as the study sample, is mainly based on argument about generalizability, as the threshold value is for reimbursement decision at national level, which affects everyone in the country. Second, it is relatively easy to enrol enough respondents among general population than other specific groups, i.e., patient group. Thus, this sample type requires less effort to select which might be favor by LMICs. Accordingly, for generalizability and feasibility, we would recommend using the general population as a study sample.

### Sample size

The sample size varies across studies, from below 100 to above 1000 respondents. However, only 9.4% of the studies (n = 6) [42, 47, 51, 84–86] gave a rationale about their sample size, and only three of them [42, 51, 85] presented the formula for their sample size calculation. As a rule of thumb, some researchers recommended that sample sizes larger than 30 and less than 500 are appropriate for most research [87–89]. However, it is also recommended that a good maximum sample size is approximately 10% of the population, as long as this does not exceed 1,000 [90, 91]. Further research is needed to investigate this issue. It is difficult for us to recommend any specific sample size; it all depends on the study setting. An ideal sample size should, however, be sufficiently large to allow the researchers to estimate reliable results [92].

### Mode of administration

Face-to-face interviews (40.6%) and web-based surveys (39.1%) were the most frequently applied modes of administration. Different modes of administration might affect the study results as well [8]. However, given the complexity of the task, we would recommend face-to-face interviews, if possible, as it enhances understanding and interactions between the interviewers and the respondents; nevertheless, it is also more resource demanding. Digital communication tools such as Skype or Zoom might be considered to reduce travel costs or other related factors.

### Hypothetical scenarios

Regarding the context of the hypothetical scenario, there is no strong evidence that one method is favored over the other (37.5% vs 42.2%). In line with previous studies, threshold values from the ex ante might be higher than those from the ex post [47, 56, 59]. Some have argued that ex ante may lead to higher uncertainty than ex post, as the ex post respondents consider other factors, such as income [47]. The ex ante is generally appropriate for identifying preferences in the case of a life-threatening disease [47]. However, in deciding whether to use ex ante or ex post scenarios for setting up a hypothetical scenario, one needs to evaluate carefully, together with other factors.

Regarding the type of hypothetical scenario, most studies were not specific to any disease/illness (62.5%), and the arguments concern its easy implementation. For those studies that applied a specific disease in the hypothetical scenario, the threshold value was positively associated with disease severity; for example, a severe cancer scenario would lead to higher threshold values [6, 51, 93] than mild ones, such as facial reanimation [34, 36]. Further investment is needed to determine whether multiple threshold values should be applied within a country, i.e., according to the disease severity or specific population, such as children. The disadvantage of a single threshold value is that for patients with severe disease such as cancer or acute or fatal diseases, it is less likely that the relevant treatment will not be reimbursed, as the relevant treatment costs are high. Therefore, it might be reasonable to consider having multiple thresholds within a country. However, this must be balanced with local health budget setting.

### Type of QALY gain

The type of QALY gain largely impacts the threshold value, with the life-saving scenario giving the highest value, followed by the life extension scenario and the quality of life improvement scenario [44, 59, 94]. The above findings may support the establishment of different thresholds for different health scenarios. In some countries, such as England and the Netherlands, separate higher thresholds for end-of-life treatments are applied [95–97]. We recommend investigating different life scenarios when eliciting thresholds in a country.

For informed QALY gain, the respondents will be informed about the magnitude or size of QALY; for uninformed QALY gain, the respondents will not be informed about the size of QALY gain. The former is applied more than the latter (64% versus 31%). WTP varies reversely with the magnitude of QALY gain [3, 16, 17, 59], and higher WTP was associated with smaller QALY gain [22, 42, 45, 98].

### Payment vehicle

Regarding payment, lump sum payment and paying in installments were the most frequently applied methods; however, nearly one-fifth of the studies did not report which method was applied. Different methods of payment vehicles may also impact the threshold values, although the pay in installment method might be associated with a higher threshold value relative to the pay in lump sum method, as the former allows respondents to pay more than once to avoid facing ceiling effect later [29, 30, 37, 39]. The choice of payment vehicle, however, needs to fit the context in the country, i.e., remain in line with the payment/reimbursement system for health care.

### Health preference-eliciting methods

For methods estimating the health preference score, the direct methods have gained popularity over the PBM (43.8% vs. 34.4%). Among the direct methods, it is most popular to apply a rating scale, either alone or mixed with SG or TTO. However, it is arguable whether the rating scale is appropriate for eliciting health utility, as it is not a choice-based method [61]. SG or TTO may be considered, as these methods are choice based and are more often recommended by economists compared to VAS [61]. However, it might be challenging to ask those questions, which is why PBMs are often applied to bypass the SG and TTO for estimating health utility [61]. Among the applied PBMs, the EQ-5D instrument was the most popular (29.7%) because it is available in many different language versions, including for LMICs, and local tariffs or neighboring countries’ tariffs might be available [99]. To estimate the health preference score, we would recommend that the researcher first check if there is any PBM available in the local language and whether a local tariff or neighbouring country is also available.

### Willingness to pay-eliciting methods (WEM)

The stated preference method was the most common choice for obtaining threshold values. Relative with this method, the reveal preference method requires data from actual behavior to derive values for health gain [62, 63] which may not be available systematically in LMICs. Meanwhile, the state preference is easier to include a wide range of scenarios, thus requiring a smaller sample size and fewer resources for conducting the study, which could be favored by LMIC. However, when using hypothetical scenarios, respondents might face challenges in imagining all the relevant components of all the scenarios, including hypothetical conditions, severity, reached outcomes, risks, or duration of scenarios [59]. Therefore, one must bear in mind that the hypothetical scenario should be carefully constructed and with proper guidelines so that the respondents can understand their task well and give reliable answers.

Regarding the WTP eliciting method, the contingent valuation method dominates (89.1%), although it contains a wide range of different approaches, such as open-ended questions, bidding games, and card sorting. Many researchers (43.8%) would mix at least two methods, usually an open-ended question with some other contingent valuation methods, to obtain a more reliable estimation. DCE has become more popular recently as it might be easy to understand for the respondent [3], decreasing the cognitive burden and the complexity of the survey, as well as the measurement error [100]. However, the design of the DCE task is rather complex, and it is challenging to evaluate whether the design has reached sufficient efficiency [101]. Using DCE to elicit stated preference, the choices are only defined by the WTP measure without involving health preference, and this method does not account for individual preference heterogeneity [102]. For LMICs, we would be more encouraged to use contingent valuation methods to elicit WTP in real situations.

### WTP/QALY combination method

There are two methods for combining WTP and QALY: the aggregated and disaggregated approaches, where the latter tends to generate higher threshold values than the former [59, 103]. The advantage of the disaggregated approach is that all individuals’ WTP for a QALY gain is imputed directly into the calculation of the mean value, but the analysis will exclude the non-traders (their WTP is 0) and respondents expressing a QALY gain of zero. The advantage of the aggregated method is its simplicity and inclusion of all respondents. However, this method does not consider the heterogeneity in preferences across individuals. In fact, some authors support the aggregated methods because of the internal consistency properties (the problem of zeros), while others account for individual WTP per QALY ratios [103]. We recommend that the choice of analysis be considered carefully, as it must be suitable for the characteristics of the data collected [104].

### Relation between WTP/QALY and GDP per capita

We also found that values for WTP/QALY were below 1 x GDP per capita in most countries despite the county’s income level. This might suggest that the WHO recommendation of applying 1–3 GDP is inappropriate, which might lead to a budget deficit because treatments could be reimbursed due to an overly high threshold. A specific high threshold could, for example, be considered cases of severe diseases or terminal illness; however, this should be introduced with clear standards/criteria and justifications to avoid funding detriment [59, 95].

### Strength and limitations

This systematic review provided a comprehensive and in-depth investigation of existing studies eliciting WTP per QALY from the direct approach and compared the existing threshold value with the WHO recommended value. Our research work provided deep insights into the different methods applied to eliciting WTP/QALY, as well as key points to consider when conducting such studies, especially in the context of LMICs. To the best of our knowledge, our study was the first with a comprehensive synthesis of the method, relevant characteristics and results of studies that elicited WTP per QALY. The application of BMA accounts for the uncertainty in variable selection by averaging over the best models, in contrast with the traditional model building strategies such as the stepwise methods, which may result in biased estimates and overly narrow confidence intervals [74].

There are, however, a few limitations need to be addressed. First, some studies did not report the time when the research was conducted, and we used the publication year instead. Sample size was evaluated based on the total sample size of the study, not the sample size of each value of WTP per QALY. Furthermore, as our recommendation was mostly based on studies from high- and upper middle-income countries (97%), a cautious need to be taken to perform WTP/QALY studies in low middle- and low-income countries, and further investigations are needed to better understand the WTP/QALY in the above context.

## Recommendations for LMICs

The utilization of the demand-side direct approach (WTP/QALY) may offer a more practical means of establishing a national threshold value within LMICs, primarily due to resource constraints and data limitations. However, this approach should be employed with thoughtful assessment of its methodological precision, applicability, and ethical consequences. Collaborative endeavors involving policymakers, researchers, and stakeholders are encouraged to establish strong and acceptable cost-effectiveness thresholds using the WTP/QALY direct approach in LMICs. To better understand the methodological barriers associated with performing WTP per QALY in LMICs, especially in low-income countries, more studies are needed in those countries. Qualitative studies, in particular, focusing on how the respondents answer the relevant questions and the stakeholder’s view about threshold value in those countries, hold particular significance and warrant further investigation. Based on findings from this review, we recommend that:

- A societal perspective might be more theoretically convincing for estimating threshold value.
- The general population shall be applied for eliciting national threshold value.
- A sufficiently large sample size that allows the researchers to estimate reliable results.
- Face-to-face interviews are recommended for mode of administration.
- The hypothetical scenario shall not be limited to any specific disease; whether using ex ante or ex post scenario one shall evaluate carefully, together with other factors.
- Different life scenarios (life-saving scenario, life extension scenario, quality of life improvement scenario) should be investigated.
- The choice of payment vehicle should depend on the context in the country (i.e., which is the most in line with the payment/reimbursement system for health in that country).
- PBMs are recommended for eliciting health preferences, given that a PBM is available in the local language and a local tariff or neighbouring country is also available. If PBM is not available, either SG or TTO can be considered.
- The combination of at least two contingent valuation method(s) is recommended, usually an open-ended question with some other contingent valuation methods, to obtain more reliable estimations.
- The choice of WTP/QALY combination method should be considered carefully, which should be suitable for the characteristics of the data collected.
- The collaborative efforts involving policymakers, researchers, and stakeholders are vital to establish robust and widely accepted thresholds.

## Conclusions

Methods for deriving WTP/QALY vary largely across studies. Eleven influential factors were identified that contribute most to the level of WTP/QALY value, in which the discrete-choice experiment method had the greatest effect. In most countries, values for WTP/QALY were below GDP per capita; therefore, in case research has not been done, the threshold suggested for LMICs is located around under GDP per capita. Some important principles are addressed related to what LMICs may be concerned with when conducting studies to estimate WTP/QALY.

## Supporting information

S1 Text Fig 1 interpretation. (DOCX)

S2 Text Detailed search terms in different databases. (DOCX)

S3 Text Full-text extraction form. (DOCX)

S4 Text Subgroup analysis. (DOCX)

S1 Table Appraisal tool for cross-sectional studies (AXIS tool). (DOCX)

S2 Table Summarized results of willingness to pay per quality-adjusted life year in 64 studies. (DOCX)

S3 Table Summarized values of willingness to pay per quality-adjusted life year by country after being converted into i$ in 2021 and WTP per QALY per GDP per capita. (DOCX)

S4 Table Summary analysis of quality appraisal of studies using the AXIS tool. (DOCX)

S1 Checklist PRISMA checklist. (DOCX)
