# Supplementary material for: A systematic review on the direct approach to elicit the demand-side cost-effectiveness threshold: Implications for low- and middle-income countries
Source: PLoS One. 2024 Feb 8;19(2):e0297450. doi: 10.1371/journal.pone.0297450 (PMC10852300; doi:10.1371/journal.pone.0297450)
Supplement: S1 Text — (DOCX) [file pone.0297450.s001.docx]

# S1 Text. Fig 1 interpretation

In terms of estimating health gain in step 1, health preference can be elicited by either using a health preference measure (direct method) or via multi-attribute utility measures (indirect method) [60, 61]. There are three main methods for directly measuring health preferences: the rating scale (RS), the time trade-off (TTO) and the standard gamble (SG) method [59, 60]. The RS method is less favoured by economists, as it is not a choice-based method [59, 60]. However, as directly measuring health preferences is a rather time consuming and complex task, an alternative approach is to bypass the measurement task by using one of the prescored multi-attribute health status classification systems, which are also known as the preference-based measures (PBM), such as the EQ-5D, SF-36 and Health Utilities Index Mark (HUI) [59]. The respondents can be the general population, patient or patient’s family member, clinicians, or mixed group.

In terms of estimating WTP in step 2, two methods can be applied: the reveal preference method, which uses data from actual behavior to derive values for health gain [61, 62], and the stated preference method, which relies on the stated behavior of individuals in a hypothetical setting [62, 63]. The most frequently used approach is the stated preference method (SP), which includes discrete choice experiments (DCE) and contingent valuation [1, 64]. The two approaches are similar in structure, but choices are offered differently. For the DCE task, the respondents are presented with a certain scenario among a series of hypothetical scenarios and are asked to state their preferred choice. Each scenario is a combination of variables (or attributes), each of which might have a number of levels. Based on the combination of attributes and the extent of these attributes in the preferred scenario of the participant, the WTP was determined [59, 64]. For contingent valuation, the choice is a bundle of different attributes where the price level varies [64]. Different techniques are used on contingent valuation, mainly bidding games, payment cards, dichotomous choices, and open-ended questions [65, 66]. In the bidding game, respondents are randomly assigned a particular bid from a range of predetermined bids and then are asked to say *yes* or *no* to that particular bid, and the process continues until the highest positive response is reached [59, 65, 67]. With payment cards, the respondents are presented with a set of offers in a table where individuals can mark out the amount that corresponds to their WTP [65, 67]. With dichotomous choice, it is either single-bounded dichotomous choice, (respondents only make a judgment as “*yes”* or “*no”* to a given amount) [67], or double-bounded dichotomous (except the yes/no question, the respondent will also be followed-up by a bid which is lower than the starting bid if their answer is “No”, or a higher follow-up bid if their answer is “Yes”) are used [67]. With open-ended questions, the respondents are directly asked what is the maximum amount they are willing to pay for a given good or service [65, 67].

Step 3 can be applied using an aggregated approach, in which WTP per QALY is estimated by dividing the average WTP by the average QALY gain from all samples, or a disaggregated approach, which estimates WTP per QALY by taking the mean of ratios of WTP per QALY elicited in each sample [2, 57].
