# Supplementary material for: A systematic review on the direct approach to elicit the demand-side cost-effectiveness threshold: Implications for low- and middle-income countries
Source: PLoS One. 2024 Feb 8;19(2):e0297450. doi: 10.1371/journal.pone.0297450 (PMC10852300; doi:10.1371/journal.pone.0297450)
Supplement: S2 Text — (DOCX) [file pone.0297450.s002.docx]

# S2 Text. Detailed searching terms in different databases

**1. PubMed**

#1 "willingness to pay"[MeSH Terms] (0)

#2 "willingness to pay"[All Fields] OR "WTP"[All Fields] (7750)

#3 "Quality-Adjusted Life Years"[Mesh] (14483)

#4 "quality adjusted life year*"[All Fields] OR "qaly*"[All Fields] (22827)

#5 #3 AND #4 (22827)

#6 #2 AND #5 (3346)

Filters: from 2000 – 2022 (3311)

**2. EMBASE**

#1 ('willingness to pay*' OR 'wtp*' OR 'willingness to pay'/exp) AND [2000-2022]/py (14067)

#2 ('quality-adjusted life year*' OR qaly* OR 'quality-adjusted life years'/exp) AND [2000-2022]/py (36697)

#3 #1 AND #2 (6101)

**3. Psycinfo (APA PsycInfo) Limiters - Publication Year: 2000-2022;**

#1 MA willingness to pay OR TX willingness to pay* OR TX WTP* (2827)

#2 MA quality adjusted life year OR TX quality adjusted life year* OR TX QALY* (3477)

#3 #1 AND #2 (247)

**4. Centre for Reviews and Dissemination (CRD)**

#1 (willingness to pay*) OR (WTP*) IN DARE, NHSEED, HTA FROM 2000 TO 2022 (897)

#2 (quality adjusted life year*) OR (QALY*) IN DARE, NHSEED, HTA FROM 2000 TO 2022 (5069)

#3 MeSH DESCRIPTOR Quality-Adjusted Life Years EXPLODE ALL TREES (3547)

#4 #2 OR #3 (5235)

#5 #4 AND #1 (691)

**5. CINAHL (Limiters - Published Date: 20000101-20220318)**

#1 MH willingness to pay OR TX willingness to pay* OR TX WTP* (13219)

#2 MH quality adjusted life years OR TX quality-adjusted life year* OR TX QALY* (10735)

#3 #1 AND #2 (1657)

**6. Econlit Limiters - Published Date: 2000 -03/2022**

#1 TX willingness to pay* OR TX WTP* (6539)

#2 TX quality adjusted life year* OR TX QALY* (650)

#3 #1 AND #2 (124)

**7. International HTA database**

#1 ((willingness to pay*) OR (WTP*)) FROM 2000 TO 2022 (87)

#2 (quality adjusted life year*) OR (QALY*) FROM 2000 TO 2022 (321)

#3"Quality-Adjusted Life Years"[mh] (78)

#4 #2 OR #3 (369)

#5 #4 AND #1 (61)
