# Supplementary material for: A systematic review on the direct approach to elicit the demand-side cost-effectiveness threshold: Implications for low- and middle-income countries
Source: PLoS One. 2024 Feb 8;19(2):e0297450. doi: 10.1371/journal.pone.0297450 (PMC10852300; doi:10.1371/journal.pone.0297450)
Supplement: S3 Text — (DOCX) [file pone.0297450.s003.docx]

# S3 Text. Full-text extraction form

(i) Publication year: Before 2000, During 2000-2005, During 2006-2010, During 2011-2015, During 2016-2020, 2021 - now;

(ii) Reporting year: Before 2000, During 2000-2005, During 2006-2010, During 2011-2015, During 2016-2020, 2021 – now, not reported;

(iii) Region: Europe, The US, Asia, Australia;

(iv) Number of countries per study: 1, >1, not reported;

(v) Type of income: High-income, Upper-middle-income, Lower-middle-income;

(vi) First author affiliation: Academic/university, Research agency/group, Government institution, not reported;

(vii) Funding source reported: Declared funding source, Declared no funding source, Did not report funding source, not reported;

(viii) Conflict of interest reported: Reported conflict of interest, Reported no conflict of interest, Did not report conflict of interest, not reported;

(ix) Perspectives: Individual, Societal, Healthcare provider, Family member of patient, Individual and societal, Individual and healthcare provider;

(x) Sample respondents: General population, Patients, Clinicians, General population and Patients, Both clinicians and politicians, Family member of patients;

(xi) Sample size: <100, 100-500, 501-1000, >1000, not reported;

(xii) Data collection method: Face-to-face interview, Telephone, Other combination, Web-based survey, Self-administered questionnaire, Secondary data analysis;

(xiii) Number of scenarios: 1, 2-5, 6-10, >10, not reported;

(xiv) Context of hypothetical scenario: Ex post: health gain treatment (worst health to better), Ex ante: Avoid health loss treatment (good health to worse), both ex post and ex ante, Not applicable/not reported;

(xv) Type of hypothetical scenario: Specific, Not specific to any diseases/illness, both specific and current health state, Not applicable/not reported;

(xvi) Type of QALY gain: Improving quality of life, extending life, saving life, improving quality of life and extending life, and improving quality of life, extending life and saving life, Others, Not applicable;

(xvii) Informed QALY gain: Informed QALY gain, Uninformed QALY gain, Not applicable;

(xviii) Duration of hypothetical scenario: < 1 month, 1 month – 1 year, > 1 year, Both duration, Not applicable/not reported;

(xix) Payment vehicle: Pay lump sum, Pay in installments, Both pay lump sum and in installments, Pay through taxes and in installments, None, Not clearly stated;

(xx) Regression analysis: Yes, No, not reported;

(xxi) Willingness-to-pay eliciting methodology: Discrete-choice experiment, Revealed preference, Contingent *(OE (Open-ended), Close-ended, Bidding game, CS (Card sorting), Payment card, SBDC (single-bound dichotomous choice), DBDC (double-bound dichotomous choice), BG and DBDC, BG and Payment cards, BG, followed by OE, CS, OE, Payment scale, OE, DBDC, OE, Others, PC, OE)*;

(xxii) Health preference eliciting methodology: Methods for directly elicit health preference (SG; TTO; VA; Person Trade-Off (PTO); SG and TTO; SG, TTO and VAS; TTO and VAS; VAS and SG; VAS and SG and TTO; TTO and rating scales), Multiattribute health preference classification system ((EQ-5D-3L (Either EQ-5D index value or EQ VAS score are used, but not specified in the study; EQ-5D index; Both EQ*-*5D index value and EQ VAS score are used), EQ-5D-5L (Either EQ-5D index value or EQ VAS score are used, but not specified in the study; EQ-5D index), EQ-5D (not specified 3L or 5L) (Either EQ-5D index value or EQ VAS score are used, but not specified in the study; EQ-5D index; Both EQ-5D index value and EQ VAS score are used) and SF-6D)), Mix of the preference-based quality of life measures (PBM) (Quality of Well-being Scale-self-administered version (QWB-SA) and EQ-5D; EQ-5D and SF-6D), Mix of the direct health preference eliciting method and PBM (EQ-5D and TTO; EQ-5D-3L and TTO and SG; SF36 and SF12 and SG and TTO and VAS), not reported;

(xxiii) WTP/QALY combination method: Aggregated, Disaggregated, Combined aggregated and disaggregated, Regression, Others, Not clearly stated;

(xxiv) Currency;

(xxv) WTP per QALY values
