# Supplementary material for: A systematic review on the direct approach to elicit the demand-side cost-effectiveness threshold: Implications for low- and middle-income countries
Source: PLoS One. 2024 Feb 8;19(2):e0297450. doi: 10.1371/journal.pone.0297450 (PMC10852300; doi:10.1371/journal.pone.0297450)
Supplement: S4 Text — (DOCX) [file pone.0297450.s004.docx]

# S4 Text. Subgroup analysis

The results of the subgroups of WTP per QALY converted in international dollars in 2021 are also illustrated in Tables 1-4. Considering WTP per QALY results between groups in case of eliminating groups of “not reported”, the results showed that the mean WTP per QALY data were the highest among studies with the year of publication during 2006-2010, reporting year during 2016-2020, conducted in Australia region, eliciting in multicountries, from high-income countries, affiliation of first author from research agency/group, having funding source, not reporting conflict of interest, perspective of family member of patient, having participants being clinicians, the sample size under 100, selecting the combination of mode of administration, and the number of hypothetical scenarios from 2 to 5. Similarly, studies had the largest mean values if they had the type of QALY gain as saving life, the context of hypothetical scenario of ex-ante, not being specific to any diseases/illness, the duration < 1 month, kind of WEM being discrete and kind of UEM being indirect method, selecting payment in installments, using fixed QALY gain, and did not use regression approach to estimation. The median WTP per QALY data had the highest values in the same groups; however, some factors had the highest median values including the affiliation of first author from government institution, having reporting conflict of interest, the number of hypothetical scenarios over 10, and the duration over 1 year.

In contrast, the lowest mean values were among studies with the year of publication year and reporting year before 2000, conducted in the US, from lower-middle-income countries, affiliation of first author from academic/university, having no funding source, reporting conflict of interest, perspective of societal inclusive individual, having participants including both general population and patients, the sample size from 100-500, selecting self-administered questionnaire, and the number of hypothetical scenarios being only one. Similarly, studies had the lowest mean values if they had the context of hypothetical scenario including both ex-ante and ex-post, being both specific and current health state, not reporting or not applicable duration of hypothetical scenario, having 2 type of WEM and 2 types of UEM, kind of WEM being other mixed method and kind of UEM being direct method, selecting payment through taxes and in installments, not using QALYs as utility measurement, and the approach of combined aggregated and disaggregated estimation. In general, WTP per QALY between groups was statistically significant (p<0.05). The median WTP per QALY data had the highest values in the same groups; however, some factors had the highest median values including the year of publication year after 2021, from upper-middle-income countries, no conflict of interest, and the number of hypothetical scenarios from 6 to 10, being specific to any diseases/illness.
