# Supplementary material for: A systematic review on the direct approach to elicit the demand-side cost-effectiveness threshold: Implications for low- and middle-income countries
Source: PLoS One. 2024 Feb 8;19(2):e0297450. doi: 10.1371/journal.pone.0297450 (PMC10852300; doi:10.1371/journal.pone.0297450)
Supplement: S1 Table — (DOCX) [file pone.0297450.s005.docx]

# S1 Table. Appraisal tool for Cross-Sectional Studies (AXIS tool)

| No. | Questions to Consider | Relevant to the study | | | | |
| --- | --- | --- | --- | --- | --- | --- |
|  |  | Yes | Partly | No | Not applicable (N/A) |  |
| Introduction | | | | | | |
| 1 | Were the aims/objectives of the study clear? |  |  |  |  |  |
| Methods | | | | | | |
| 2 | Was the study design appropriate for the stated aim(s)? |  |  |  |  |  |
| 3 | Was the sample size justified? |  |  |  |  |  |
| 4 | Was the target/reference population clearly defined?  (Is it clear who the research was about?) |  |  |  |  |  |
| 5 | Was the sample frame taken from an appropriate population base so that it closely represented the target/reference population under investigation? |  |  |  |  |  |
| 6 | Was the selection process likely to select subjects/participants that were representative of the target/reference population under investigation? |  |  |  |  |  |
| 7 | Were measures undertaken to address and categorize non-responders? |  |  |  |  |  |
| 8 | Were the risk factor and outcome variables measured appropriate to the aims of the study? |  |  |  |  |  |
| 9 | Were the risk factor and outcome variables measured correctly using instruments/measurements that had been trialed, piloted or published previously? |  |  |  |  |  |
| 10 | Is it clear what was used to determined statistical significance and/or precision estimates? (e.g., p values, Cis) |  |  |  |  |  |
| 11 | Were the methods (including statistical methods) sufficiently described to enable them to be repeated? |  |  |  |  |  |
| Results | | | | | | |
| 12 | Were the basic data adequately described? |  |  |  |  |  |
| 13 | Does the response rate raise concerns about non-response bias? |  |  |  |  |  |
| 14 | If appropriate, was information about non-responders described? |  |  |  |  |  |
| 15 | Were the results internally consistent? |  |  |  |  |  |
| 16 | Were the results for the analyses described in the methods, presented? |  |  |  |  |  |
| 17 | Were the authors’ discussions and conclusions justified by the results? |  |  |  |  |  |
| 18 | Were the limitations of the study discussed? |  |  |  |  |  |
| Others | | | | | | |
| 19 | Were there any funding sources or conflicts of interest that may affect the authors’ interpretation of the results? |  |  |  |  |  |
| 20 | Was ethical approval or consent of participants attained? |  |  |  |  |  |
