# Supplementary material for: A systematic review on the direct approach to elicit the demand-side cost-effectiveness threshold: Implications for low- and middle-income countries
Source: PLoS One. 2024 Feb 8;19(2):e0297450. doi: 10.1371/journal.pone.0297450 (PMC10852300; doi:10.1371/journal.pone.0297450)
Supplement: S2 Table — (DOCX) [file pone.0297450.s006.docx]

# S2 Table. Summarized results of willingness to pay per quality-adjusted life year in 64 studies

| **Reference** | **Publication year** | **Reporting year** | **Currency** | **Country** | **WTP/QALY (original)** | **WTP/QALY**  **(USD)** | **WTP/QALY  per GDP/capita** | **Converted WTP/QALY (i$)** |
| --- | --- | --- | --- | --- | --- | --- | --- | --- |
| (74) | 2018 | n/a | AUD | Australia | 42,250.0 - 42,250.0 | 31,429.8 - 31,429.8 | 0.5 – 0.5 | 30,417.9 -30,417.9 |
| (6) | 2019 | 2018 | Euro | Bulgaria | 93,500.0 - 96,186.0 | 110,152.4 - 113,316.7 | 11.7 – 12.0 | 143,768.7 - 147,898.8 |
| (48) | 2015 | 2012 | USD | China | 1,740.4 - 9,446.1 | 1,740.4 - 9,446.1 | 0.3 - 1.5 | 1,959.1 - 10,633.3 |
| (47) | 2011 | 2009 | USD | China | 4,711.0 - 7,408.0 | 4,711.0 - 7,408.0 | 1.2 - 1.9 | 6,288.0 - 9,887.8 |
| (75) | 2021 | 2019 | RMB | China | 7,500.0 - 500,000.0 | 1,086.8 - 72,450.0 | 0.1 - 7.1 | 1,935.8 - 129,051.3 |
| (76) | 2003 | 2001 | DKK | Denmark | 88,000.0 - 88,000.0 | 10,533.6 - 10,533.6 | 0.3 - 0.3 | 18,295.2 - 18,295.2 |
| (77) | 2012 | 2005 | DKK | Denmark | 148,900.0 - 203,485.0 | 24,747.2 - 33,819.2 | 0.5 - 0.7 | 28,748.7 - 39,287.6 |
| (78) | 2021 | 2009-2010 | Euro | Denmark | 35,598.0 - 35,598.0 | 47,025.0 - 47,025.0 | 0.8 - 0.8 | 6,186.4 - 6,186.4 |
| (79) | 2017 | 2013 | Euro | Denmark | 2,805.0 - 85,405.0 | 3,730.1 - 113,571.6 | 0.1 - 2.0 | 459.6 - 13,995.0 |
| (7) | 2012 | 2006 | Euro | Finland | 13,847.0 - 102,616.0 | 20,371.7 - 150,968.7 | 0.5 - 3.7 | 21,025.1 - 155,811.0 |
| (9) | 2016 | 2012-2014 | Euro | Germany | 8,580.0 - 18,420.0 | 11,334.2 - 24,332.8 | 0.2 - 0.5 | 13,179.4 - 28,294.3 |
| (8) | 2013 | 2012 | Euro | Germany | 7,473.0 - 195,040.0 | 7,473.0 - 195,040.0 | 0.2 - 4.4 | 8,396.4 - 219,140.3 |
| (10) | 2021 | 2019 | Euro | Germany | 2,892.0 - 7,676.0 | 3,232.1 - 8,578.7 | 0.1 - 0.2 | 4,078.0 - 10,824.0 |
| (12) | 2020 | 2019 | Euro | Greece | 26,279.9 - 26,279.9 | 29,370.4 - 29,370.4 | 1.5 - 1.5 | 48,784.5 - 48,784.5 |
| (11) | 2017 | 2016 | Euro | Greece | 960.0 - 1,536.0 | 1,059.3 - 1,694.8 | 0.1 - 0.1 | 1,817.9 - 2,908.7 |
| (41) | 2020 | 2019 | USD | Iran | 1,101.0 - 1,355.0 | 1,101.0 - 1,355.0 | 0.4 - 0.4 | 1,240.8 - 1,527.1 |
| (37) | 2017 | 2014-2015 | USD | Iran | 48,350,730.0 -64,734,470.0 | 1,160.4-1,553.6 | 0.24-0.32 | 3,328.9 - 4,456.9 |
| (39) | 2019 | 2015 | USD | Iran | 2,378.0 - 5,043.0 | 2,378.0 - 5,043.0 | 0.5 - 1.0 | 5,127.4 - 10,873.6 |
| (40) | 2019 | 2015 | USD | Iran | 305.0 - 2,666.0 | 305.0 - 2,666.0 | 0.1 - 0.5 | 657.6 - 5,748.4 |
| (38) | 2018 | 2017 | USD | Iran | 1,008.0 - 4,773.0 | 1,008.0 - 4,773.0 | 0.2 - 0.9 | 1,875.7 - 8,881.6 |
| (80) | 2021 | n/a | USD | Iran | 6,694.9 - 7,287.1 | 6,694.9 - 7,287.1 | 2.8 - 3.0 | 6,694.9 - 7,287.1 |
| (81) | 2013 | n/a | USD | Israel | 80,299.0 - 195,040.0 | 80,299.0 - 195,040.0 | 2.2 - 5.4 | 81,996.3 - 199,162.6 |
| (45) | 2013 | 2011 | JPY | Japan | 1,810,000.0 - 9,050,000.0 | 22,784.5 - 113,922.5 | 0.5 - 2.4 | 19,733.9 - 98,669.7 |
| (46) | 2019 | 2018 | JPY | Japan | 2,600,000.0 -14,900,000.0 | 23,576.4 - 135,110.6 | 0.6 - 3.5 | 26,929.7 - 154,328.1 |
| (82) | 2017 | 2014 | MYR | Malaysia | 12,810.0 - 22,840.0 | 3,905.5 - 6,963.4 | 0.3 - 0.6 | 9,104.1 - 16,232.4 |
| (49) | 2017 | 2012-2014 | MYR | Malaysia | 6,200.0 - 8,900.0 | 1,890.2 - 2,713.4 | 0.2 - 0.2 | 4,406.3 - 6,325.2 |
| (83) | 2014 | 2010 | MYR | Malaysia | 24,942.0 - 24,942.0 | 7,771.9 - 7,771.9 | 0.9 - 0.9 | 19,581.3 - 19,581.3 |
| (13) | 2013 | n/a | Euro | Netherlands | 119,600.0 - 188,900.0 | 159,044.1 - 251,199.2 | 3.0 - 4.8 | 176,949.7 - 279,479.9 |
| (14) | 2015 | 2013 | Euro | Netherlands | 184,893.0 - 209,588.0 | 245,870.7 - 278,710.1 | 4.7 - 5.3 | 273,551.5 - 310,088.1 |
| (3) | 2010 | 2008 | Euro | Netherlands | 12,900.0 - 24,500.0 | 18,978.5 - 36,044.4 | 0.3 - 0.6 | 21,022.6 - 39,926.7 |
| (15) | 2012 | 2008 | Euro | Netherlands | 6,800.0 - 16,200.0 | 10,004.2 - 23,833.4 | 0.2 - 0.4 | 11,081.7 - 26,400.5 |
| (84) | 2016 | n/a | Euro | Netherlands | 227,200.0 - 404,400.0 | 300,040.3 - 534,050.6 | 5.7 - 10.2 | 336,145.2 -598,314.9 |
| (85) | 2013 | n/a | Euro | Netherlands | 250,500.0 - 250,500.0 | 333,114.9 - 333,114.9 | 6.4 - 6.4 | 370,617.9 - 370,617.9 |
| (86) | 2015 | n/a | Euro | Netherlands | 17,824.0 - 17,824.0 | 23,702.4 - 23,702.4 | 0.5 - 0.5 | 14,912.0 - 14,912.0 |
| (87) | 2021 | 2019 | Euro | Netherlands | 87.6 - 100.8 | 97.9 - 112.7 | 0.0 - 0.0 | 120.2 - 138.3 |
| (88) | 2021 | 2019 | Euro | Netherlands | 104.4 - 104.4 | 116.7 - 116.7 | 0.0 - 0.0 | 143.2 - 143.2 |
| (89) | 2018 | 2015 | KRW | Korea | 33,528,000.0 - 37,758,000.0 | 29,531.5 - 33,257.3 | 1.0 - 1.2 | 52,057.0 - 58,624.7 |
| (16) | 2015 | 2010 | PPP | Netherland The UK France Spain Sweden Norway Denmark Poland Hungary Palestine | 10,709.0 - 29,062.0 | 10,709.0 - 29,062.0 | 0.2 - 0.6 | 13,307.7 - 36,114.3 |
| (19) | 2019 | 2016-2017 | Euro | Spain | 9,795.0 - 25,503.0 | 11,156.5 - 29,047.9 | 0.4 - 1.0 | 16,765.6 - 43,652.1 |
| (17) | 2009 | n/a | Euro | Spain | 8,151.0 - 123,724.0 | 11,369.0 - 172,570.2 | 0.4 - 5.4 | 15,392.3 - 233,639.2 |
| (90) | 2014 | n/a | Euro | Spain | 10,119.0 - 28,187.0 | 13,363.2 - 37,223.8 | 0.5 - 1.3 | 19,053.6 - 53,074.8 |
| (91) | 2021 | n/a | Euro | Spain | 11,473.0 - 19,092.0 | 13,534.7 - 22,522.8 | 0.5 - 0.8 | 18,664.8 - 31,059.8 |
| (92) | 2014 | 2010 | Euro | Spain | 371.1 - 2,083.1 | 490.2 - 2,751.8 | 0.0 - 0.1 | 688.4 - 3,864.2 |
| (22) | 2018 | 2017 | SEK | Sweden | 104,091.0 - 2,885,118.0 | 12,282.7 - 340,443.9 | 0.2 - 6.3 | 12,964.1 - 359,329.9 |
| (21) | 2017 | 2016 | SEK | Sweden | 4,800,000.0 - 4,800,000.0 | 559,200.0 - 559,200.0 | 10.8 - 10.8 | 608,548.7 - 608,548.7 |
| (93) | 2010 | 2007 | Pound | UK | 17,955.5 - 22,585.8 | 36,061.8 - 45,361.2 | 0.7 - 0.9 | 36,021.3 - 45,310.4 |
| (20) | 2013 | 2009-2010 | PPP | The UK Netherlands France Spain Norway Sweden Denmark Hungary Poland | 18,247.0 - 34,097.0 | 18,247.0 - 34,097.0 | 0.4 - 0.7 | 22,674.9 - 42,371.1 |
| (27) | 2004 | 2001 | USD | The US | 5,000.0 - 5,000.0 | 5,000.0 - 5,000.0 | 0.1 - 0.1 | 7,651.6 - 7,651.6 |
| (31) | 2010 | 2005-2006 | USD | The US | 18,000.0 - 60,000.0 | 18,000.0 - 60,000.0 | 0.4 - 1.3 | 24,194.7 - 80,649.1 |
| (33) | 2016 | 2015 | USD | The US | 2,072.0 - 16,277.0 | 2,072.0 - 16,277.0 | 0.0 - 0.3 | 2,368.8 - 18,608.7 |
| (34) | 2016 | n/a | USD | The US | 19.0 - 2,838.0 | 19.0 - 2,838.0 | 0.0 - 0.0 | 21.5 - 3,204.1 |
| (36) | 2020 | 2019 | USD | The US | 1,612.3 - 12,264.3 | 1,612.3 - 12,264.3 | 0.0 - 0.2 | 1,708.9 - 12,998.8 |
| (30) | 2009 | 2005 | USD | The US | 30,000.0 - 44,000.0 | 30,000.0 - 44,000.0 | 0.7 - 1.0 | 43,037.6 - 63,121.9 |
| (28) | 2005 | 2003 | USD | The US | 12,500.0 - 32,200.0 | 12,500.0 - 32,200.0 | 0.3 - 0.8 | 18,412.4 - 47,430.4 |
| (29) | 2005 | 2001 | USD | The US | 1,387.2 - 6,946.2 | 1,387.2 - 6,946.2 | 0.0 - 0.2 | 2,122.9 - 10,630.0 |
| (42) | 2013 | 2008 | Baht | Thailand | 28,000.0 - 285,000.0 | 840.0 - 8,550.0 | 0.2 - 2.0 | 2,361.6 - 24,038.1 |
| (43) | 2015 | n/a | USD | Thailand | 11,093.0 - 54,432.0 | 322.8 - 1,584.0 | 0.1 - 0.3 | 926.6 - 4,546.6 |
| (44) | 2015 | 2013-2014 | USD | Thailand | 243,120.0 - 244,720.0 | 7,463.8 - 7,512.9 | 1.3 - 1.3 | 20,124.4 - 20,256.8 |
| (94) | 2020 | 2017 | US dollars | The Kingdom of Saudi Arabia | 22,720.0 - 32,000.0 | 22,720.0 - 32,000.0 | 1.1 - 1.5 | 24,298.7 - 34,223.5 |
| (50) | 2020 | 2018 | USD | Vietnam | 11,301.0 - 11,301.0 | 11,301.0 - 11,301.0 | 4.4 - 4.4 | 11,879.0 - 11,879.0 |
| (95) | 2010 | 2007-2008 | Multi currency | Taiwan Japan Korea Australia UK The US | 36,000.0 - 96,000.0 | 36,000.0 - 96,000.0 | 0.7 - 2.0 | 45,307.9 - 120,821.2 |
| (96) | 2017 | 2015 | USD | The US | 3,386.0 - 18,001.0 | 3,386.0 - 18,001.0 | 0.1 - 0.3 | 3,871.0 - 20,579.6 |
| (97) | 2015 | 2013 | SGD | Singapore | 1,587.0 - 13,016.0 | 1,153.4 - 9,460.0 | 0.0 - 0.2 | 1,965.2 - 16,118.3 |
| (98) | 1998 | 1996 | SEK | Sweden | 118,400.0 - 118,400.0 | 17,641.6 - 17,641.6 | 0.5 - 0.5 | 18,460.7 - 18,460.7 |
