# Supplementary material for: A systematic review on the direct approach to elicit the demand-side cost-effectiveness threshold: Implications for low- and middle-income countries
Source: PLoS One. 2024 Feb 8;19(2):e0297450. doi: 10.1371/journal.pone.0297450 (PMC10852300; doi:10.1371/journal.pone.0297450)
Supplement: S3 Table — (DOCX) [file pone.0297450.s007.docx]

# S3 Table. Summarized values of willingness to pay per quality-adjusted life year by country after being converted into i$ in 2021 and WTP per QALY per GDP per capita

| **No** | **Country** | **WTP per QALY** | | **WTP per QALY per GDP/capital** | |
| --- | --- | --- | --- | --- | --- |
|  |  | **Median (IQR)** | **Mean (SD)** | **Median (IQR)** | **Mean (SD)** |
| 1 | Australia | 30,417.9 (0.0) | 30,417.9 (0.0) | 0.548 (0.0) | 0.548 (0.0) |
| 2 | Bulgaria | 145,833.8 (0.2) | 145,833.8 (2,920.4) | 11.852 (0.2) | 11.852 (0.2) |
| 3 | China | 9,037.7 (1.6) | 24,338.2 (33,537.5) | 0.985 (1.6) | 1.575 (1.8) |
| 4 | Denmark | 5,545.6 (0.4) | 10,604.8 (11,682.9) | 0.585 (0.4) | 0.672 (0.5) |
| 5 | EU | 27,409.4 (0.2) | 28,145.2 (10,560.5) | 0.455 (0.2) | 0.467 (0.2) |
| 6 | Finland | 63,433.7 (2.5) | 78,664.9 (62,780.3) | 1.492 (2.5) | 1.851 (1.5) |
| 7 | Germany | 18,768.0 (0.7) | 40,968.5 (56,474.9) | 0.381 (0.7) | 0.817 (1.2) |
| 8 | Greece | 2,643.6 (0.0) | 11,698.7 (20,735.6) | 0.086 (0.0) | 0.370 (0.7) |
| 9 | Iran | 3,594.3 (0.4) | 4,122.5 (2,766.7) | 0.341 (0.4) | 0.504 (0.6) |
| 10 | Israel | 109,626.8 (1.8) | 125,103.1 (54,968.2) | 2.956 (1.8) | 3.373 (1.5) |
| 11 | Japan | 48,680.7 (0.7) | 54,453.2 (28,659.9) | 1.088 (0.7) | 1.273 (0.7) |
| 12 | Malaysia | 9,104.1 (0.4) | 11,129.9 (6,516.5) | 0.345 (0.4) | 0.445 (0.3) |
| 13 | Many continents | 80,547.4 (0.5) | 78,240.1 (20,816.0) | 1.323 (0.5) | 1.285 (0.3) |
| 14 | Netherlands | 20,289.3 (4.1) | 110,700.0 (162,620.7) | 0.338 (4.1) | 1.899 (2.8) |
| 15 | Singapore | 9,865.9 (0.1) | 9,453.8 (6,579.1) | 0.102 (0.1) | 0.097 (0.1) |
| 16 | South Korea | 54,062.2 (0.0) | 54,701.5 (2,784.9) | 1.067 (0.0) | 1.080 (0.1) |
| 17 | Spain | 23,364.8 (0.7) | 29,324.0 (40,223.3) | 0.578 (0.7) | 0.702 (0.9) |
| 18 | Sweden | 80,931.2 (3.6) | 185,428.8 (220,938.9) | 1.425 (3.6) | 3.302 (3.9) |
| 19 | Thailand | 7,042.7 (0.5) | 9,144.3 (6,992.4) | 0.572 (0.5) | 0.688 (0.5) |
| 20 | The Kingdom of Saudi Arabia | 29,261.1 (0.2) | 29,261.1 (7,017.9) | 1.315 (0.2) | 1.315 (0.3) |
| 21 | UK | 40,665.9 (0.1) | 40,665.9 (6,568.3) | 0.807 (0.1) | 0.807 (0.1) |
| 22 | USA | 7,312.2 (0.4) | 15,594.3 (19,473.8) | 0.127 (0.4) | 0.257 (0.3) |
| 23 | Vietnam | 11,879.0 (0.0) | 11,879.0 (0.0) | 4.403 (0.0) | 4.403 (0.0) |
| **All values** | | **16,647.6** | **38,613.7** | **0.534** | **1.012** |
