# Supplementary material for: A systematic review on the direct approach to elicit the demand-side cost-effectiveness threshold: Implications for low- and middle-income countries
Source: PLoS One. 2024 Feb 8;19(2):e0297450. doi: 10.1371/journal.pone.0297450 (PMC10852300; doi:10.1371/journal.pone.0297450)
Supplement: S4 Table — (DOCX) [file pone.0297450.s008.docx]

# S4 Table. Summary analysis of quality appraisal of studies using the AXIS tool

| **No.** | **Questions to Consider** | **Rating study** | | | |
| --- | --- | --- | --- | --- | --- |
|  |  | **Yes** | **No** | **Un-clear** | **N/A** |
| **Introduction** | | | | | |
| 1 | Were the aims/objectives of the study clear? | 100% (N=64) | 0.00% (N=0) | 0.00% (N=0) | 0.00% (N=0) |
| **Methods** | | | | | |
| 2 | Was the study design appropriate for the stated aim(s)? | 100% (N=64) | 0.00% (N=0) | 0.00% (N=0) | 0.00% (N=0) |
| 3 | Was the sample size justified? | 9.38% (N=6) | 71.88% (N=46) | 15.63% (N=10) | 3.13% (N=2) |
| 4 | Was the target/reference population clearly defined? (Is it clear who the research was about?) | 100% (N=64) | 0.00% (N=0) | 0.00% (N=0) | 0.00% (N=0) |
| 5 | Was the sample frame taken from an appropriate population base so that it closely represented the target/reference population under investigation? | 50.00% (N=32) | 6.25% (N=4) | 34.38% (N=22) | 9.38% (N=6) |
| 6 | Was the selection process likely to select subjects/participants that were representative of the target/reference population under investigation? | 40.63% (N=26) | 3.13% (N=2) | 45.31% (N=29) | 10.94% (N=7) |
| 7 | Were measures undertaken to address and categorize non-responders? | 6.25% (N=4) | 54.69% (N=35) | 17.19% (N=11) | 21.88% (N=14) |
| 8 | Were the risk factor and outcome variables measured appropriate to the aims of the study? | 96.88% (N=62) | 3.13% (N=2) | 0.00% (N=0) | 0.00% (N=0) |
| 9 | Were the risk factor and outcome variables measured correctly using instruments/measurements that had been trialed, piloted or published previously? | 92.19% (N=59) | 0.00% (N=0) | 7.81% (N=5) | 0.00% (N=0) |
| 10 | Is it clear what was used to determined statistical significance and/or precision estimates? (e.g., p values, Cis) | 98.44% (N=63) | 1.56% (N=1) | 0.00% (N=0) | 0.00% (N=0) |
| 11 | Were the methods (including statistical methods) sufficiently described to enable them to be repeated? | 89.06% (N=57) | 0.00% (N=0) | 10.94% (N=7) | 0.00% (N=0) |
| **Results** | | | | | |
| 12 | Were the basic data adequately described? | 98.44% (N=63) | 1.56% (N=1) | 0.00% (N=0) | 0.00% (N=0) |
| 13 | Does the response rate raise concerns about non-response bias? | 10.94% (N=7) | 25.00% (N=16) | 37.50% (N=24) | 26.56% (N=17) |
| 14 | If appropriate, was information about non-responders described? | 12.50% (N=8) | 75.00% (N=48) | 3.13% (N=2) | 9.38% (N=6) |
| 15 | Were the results internally consistent? | 48.44% (N=31) | 17.19% (N=11) | 26.56% (N=17) | 7.81% (N=5) |
| 16 | Were the results for the analyses described in the methods, presented? | 95.31% (N=61) | 1.56% (N=1) | 3.13% (N=2) | 0.00% (N=0) |
| 17 | Were the authors’ discussions and conclusions justified by the results? | 92.19% (N=59) | 3.13% (N=2) | 4.69% (N=3) | 0.00% (N=0) |
| 18 | Were the limitations of the study discussed? | 81.25% (N=52) | 18.75% (N=12) | 0.00% (N=0) | 0.00% (N=0) |
| **Others** | | | | | |
| 19 | Were there any funding sources or conflicts of interest that may affect the authors’ interpretation of the results? | 10.94% (N=7) | 65.63% (N=42) | 14.06% (N=9) | 9.38% (N=6) |
| 20 | Was ethical approval or consent of participants attained? | 51.56% (N=33) | 10.94% (N=7) | 1.56% (N=1) | 35.94% (N=23) |
